# Multiparametric flow cytometry to characterize vaccine-induced polyfunctional T cell responses and T cell/NK cell exhaustion and memory phenotypes in mouse immuno-oncology models

**DOI:** 10.3389/fimmu.2023.1127896

**Published:** 2023-04-06

**Authors:** Davide Moi, Bijun Zeng, Simone A. Minnie, Rituparna Bhatt, Jack Wood, David P. Sester, Roberta Mazzieri, Riccardo Dolcetti

**Affiliations:** ^1^ The University of Queensland Frazer Institute, Woolloongabba, QLD, Australia; ^2^ Peter MacCallum Cancer Centre, Melbourne, VIC, Australia; ^3^ Clinical Research Division, Fred Hutchinson Cancer Research Center, Seattle, WA, United States; ^4^ TRI Flow Cytometry Suite, Translational Research Institute, Woolloongabba, QLD, Australia; ^5^ Sir Peter MacCallum Department of Oncology, The University of Melbourne, Melbourne, VIC, Australia; ^6^ Department of Microbiology and Immunology, The University of Melbourne, Melbourne, VIC, Australia

**Keywords:** immunomonitoring, polyfunctional T cell assay, multiparametric flow cytometry, cancer vaccines, mouse cancer models, cancer immunotherapy, immune checkpoints

## Abstract

Suitable methods to assess *in vivo* immunogenicity and therapeutic efficacy of cancer vaccines in preclinical cancer models are critical to overcome current limitations of cancer vaccines and enhance the clinical applicability of this promising immunotherapeutic strategy. In particular, availability of methods allowing the characterization of T cell responses to endogenous tumor antigens is required to assess vaccine potency and improve the antigen formulation. Moreover, multiparametric assays to deeply characterize tumor-induced and therapy-induced immune modulation are relevant to design mechanism-based combination immunotherapies. Here we describe a versatile multiparametric flow cytometry method to assess the polyfunctionality of tumor antigen-specific CD4^+^ and CD8^+^ T cell responses based on their production of multiple cytokines after short-term *ex vivo* restimulation with relevant tumor epitopes of the most common mouse strains. We also report the development and application of two 21-color flow cytometry panels allowing a comprehensive characterization of T cell and natural killer cell exhaustion and memory phenotypes in mice with a particular focus on preclinical cancer models.

## 1 Introduction

Despite significant technological advances obtained in the last decade, only a very limited number of cancer vaccines progressed to phase III clinical trials (1). A thorough and in-depth characterization of feasibility, immunogenicity and therapeutic efficacy of cancer vaccines in suitable preclinical models remains critical to overcome current limitations and enhance the clinical applicability of this promising immunotherapeutic strategy. In particular, assays allowing a reliable characterization of T cell responses to endogenous tumor antigens in mouse models is required to assess vaccine potency and improve the antigen formulation. Moreover, multiparametric assays are required to comprehensively characterize the immune modulation and regulatory/immunosuppressive effects generated by the tumor itself or induced by the therapy to design mechanism-based combination immunotherapies.

Therapeutically effective cancer vaccines should generate strong tumor-specific immune responses that need to be evaluated by measuring adequate parameters to assess vaccine potency. In this respect, the mere enumeration of T cells recognizing the antigens targeted by the vaccine is not sufficient and should be implemented by a more appropriate qualitative characterization of the functional properties of vaccine-induced CD4^+^ and CD8^+^ T lymphocytes (2, 3). Therefore, assays able to assess the polyfunctionality of antigen-specific T cells are particularly relevant to optimize the antigen formulation of cancer vaccines and assess the strength and quality of the T cell responses induced *in vivo* in naïve or tumor bearing mice. Here we describe a versatile multiparametric flow cytometry method to assess the polyfunctionality of tumor antigen-specific CD4^+^ and CD8^+^ T cell responses based on their production of multiple cytokines after short-term *ex vivo* restimulation with relevant mouse tumor epitopes. The method can be useful to characterize the quality of T cell responses induced not only by the antigens targeted by the vaccine, but also by “universal” tumor associated antigens as a measure of therapy-induced epitope spreading.

The generation of strong tumor-specific T cell responses by cancer vaccines is often not sufficient to achieve satisfactory therapeutic responses. Indeed, the efficacy of cancer vaccines and other immunotherapies is hampered by systemic and local immune suppression, which subvert anti-tumor immune responses leading to dysfunctional and exhausted immune cells. T and NK cell function is dependent on the balance between activation/co-stimulation and the consequent upregulation of inhibitory signals triggered by numerous inhibitory immune checkpoint pathways (4). This complex landscape of regulatory molecules evolves dynamically over time and may be also heavily modulated by treatment, particularly immunotherapies. As such, profiling the expression of these regulatory molecules has become a major focus to inform therapeutic choices and identify novel therapeutic targets. Moreover, the assessment of preclinical tumor microenvironments and associated lymphoid organs (spleens and lymph nodes) for changes in expression of regulatory molecules is also required to identify possible evasion mechanisms and facilitate the rationale design of more effective combination therapies (5). Finally, the strength and duration of therapeutic responses induced by immunotherapy are closely dependent on the induction of strong and long-lasting memory immune responses (6). To be able to evaluate local and systemic immune modulation, we have developed two 21-color flow cytometry panels to comprehensively characterize T cell and natural killer (NK) cell exhaustion and memory phenotypes in mice with a particular focus on preclinical cancer models.

## 2 Methods

### 2.1 Cell lines and tissue cultures

Murine triple negative breast cancer 4T1.2 cells were kindly provided by Prof. B. Parker (Peter MacCallum Cancer Centre, Melbourne, Australia). Murine melanoma B16F10 cells were kindly provided by Prof. N. Haas, University of Queensland). Murine melanoma YUMM UV 1.7 UV cells (7) (generated by UV irradiating YUMM 1.7 cells) were kindly provided by Prof. B. Gabrielli (Mater Research, University of Queensland). Cells were grown at 37°C and 5% CO_2_ and passed using TrypLE Express when reaching 80% confluence. 4T1.2 and B16F10 cells were cultured in DMEM (Gibco) containing 10% FBS (Gibco), 2mM L-Glutamine (Gibco), 100IU/ml Penicillin (Gibco), 100µg/ml Streptomycin (Gibco) and 1mM Sodium Pyruvate (Gibco). YUMM UV 1.7 UV cells were cultured in DMEM/F12 (Gibco) containing 10% FBS (Gibco), 2mM L-Glutamine (Gibco), 100IU/ml Penicillin (Gibco), 100µg/ml Streptomycin (Gibco) and 1mM Sodium Pyruvate (Gibco).

### 2.2 Vaccine preparation

*Tumor cell lysate preparation.* Eighty percent confluent tumor cells from six T75 culture flasks were harvested, washed 3 times with PBS and resuspended in 0.5 mL sterile water and then subjected to 5 cycles of freeze and thaw in liquid nitrogen. Lysates were centrifuged at 10,000 rpm for 20 min at 4°C and the recovered supernatant quantified for protein concentration using a NanoDropTM 2000 spectrophotometer and diluted to 5 mg/mL in water. Aliquots of 200 µL of lysates were converted to powder by snap-freezing in liquid nitrogen followed by overnight lyophilization (0.05 mbar, -80°C) and stored at -80°C. Lysate powder was dissolved in 1 mL ultrapure water to a final protein concentration of 1mg/mL. The tumor cell lysate solution (0.4 mL) was added to 0.8 mL of CithrolTM GMO HP solution into a 1.5 mL vial and mixed with a sonicator (Branson Sonifier^®^ S-450A, Danbury, United State) for 1 min at 20 W to form stable water in oil (w/o) emulsion. CithrolTM GMO HP solution (1%, w/v) was prepared by dissolving CithrolTM GMO HP (gift from Croda Europe Ltd) in Hexane (Sigma). The resulting emulsion was frozen in dry ice for 2 h before being lyophilized for 24 h. The lyophilized lysate-Cithrol GMO HP pellet was dissolved in Miglyol 812 (10 µL; (gift from Cremer Oleo GmbH & Co. KG) to 5mg/ml and used as oil phase for preparing Clec9A-targeting Tailored NanoEmusions (TNE) loaded with tumor cell lysate as antigen formulation (Lysate-Clec9A-TNE) as described in Zeng et al. (8) and below.

*Preparation of Clec9A-targeting Tailored NanoEmusions (TNE) loaded with tumor cell lysate.* 490 µL of AM1 (400 µM; GL Biochem, China) was added to 10 μL of lysate loaded Miglyol 812 to give an oil volume fraction of 2% (v/v). The mixture was homogenized using a Branson Sonifier 450A ultrasonicator for four 45 s bursts at 60 W. The resulting oil-in-water (O/W) emulsion was coated with PEG (200 µM; Nanocs, US) and the mouse Clec9A targeting WH peptide fused to anchor peptide DAMP4 (3 µM) by gentle mixing for 60 min at room temperature. Clec9A-TNEs loaded with B16F10 immunogenic neoepitopes were prepared as described (8).

### 2.3 Mouse studies

All animal experiments were approved by the University of Queensland Animal Ethics Committee (approval number UQDI/252/16). Mice were obtained from the Animal Resources Centre (Perth, WA, Australia).

*Murine triple negative breast cancer model*. Eight to ten-week-old female Balb/c mice were injected with 0.5×10^6^ 4T1.2 cells (50 µl) orthotopically in the 3^rd^ shaved mammary fat pad. Three days after tumor injection, mice were injected i.v. with 100 μL Clec9a-TNE loaded with the 4T1.2 lysate (Clec9A-TNE-lysate). At the indicated times tumors and/or draining lymph nodes were collected and processed as described below.

*Murine B16F10 melanoma model*. Eight to ten-week-old female C57BL/6 mice were injected with 0.25×10^6^ B16F10 cells (50 µl) subcutaneously in the shaved right back flank. Ten days after tumor injection, mice were injected i.v. with 100μL Clec9a-TNE loaded with a pool of functionally validated, immunogenic B16F10 neo-epitopes (25 μg) (8). Mice without treatment were included as controls (NT CTRL). Tumor growth was measured two to three times per week using calliper. Tumor volume was measured using an electronic caliper and calculated using the following formula: (m1)^2^ X m2 X 0.5236 – where m1 stand for the small tumor diameter, and m2 for the long one. Spleens were collected at the indicated times for processing as described below.

*Murine YUMM UV 1.7 melanoma model*. Tumor-bearing mice were generated by injecting 2x10^6^ melanoma cells (50µl) subcutaneously into C57BL/6 mice. Tumors, spleens and draining lymph nodes were collected at the indicated times for processing as described below.

The YUMM UV 1.7 and 4T1.2 mouse models were used to set up and optimize the two multiparametric flow cytometry panels. The 4T1.2 and B16F10 models were used for the polyfunctional assay on blood.

### 2.4 Polyfunctional T cell assay

*Ex vivo restimulation with tumor specific peptides*. Blood from tumour-bearing mice was collected into EDTA treated tubes and red blood cells were lysed by adding ACK (Ammonium-Chloride-Potassium) buffer after centrifugation and removal of supernatant and incubating for 5 min on ice. ACK buffer was prepared according to the Cold Spring Harbor Protocol: 150 mM NH4Cl, 10 mM KHCO3, 0.1 mM Na2EDTA. After washing with complete RPMI medium (Gibco), white blood cells were plated in complete RPMI medium (10% FBS, 2mM L-Glutamine, 100IU/ml Penicillin, 100µg/ml Streptomycin and 1mM Sodium Pyruvate) in round bottom 96-well plates at a density of 1x10^6^ cells/well and ex vivo re-stimulated for 6 hours at 37°C with the antigens/epitopes listed in **Table 1** (20 µg/mL in a total volume of 200 µL). Brefeldin A (Biolegend) was added after 1 hour to block cytokine release at a final concentration of 5 µg/ml.

**Table 1:** Tumor-specific epitope peptides for *ex vivo* restimulation of T cells.

Mouse model	Pool of peptides	Epitope	Sequence	Presenting MHC molecule	Reference
**C57Bl/6 mice bearing B16F10 melanoma**	neo-epitopes^a^	MUT20	FRRKAFLHWYTGEAMDEMEFTEAESNM	I-A^b^, H-2D^b^, H-2K^b^	Zeng, (8)
		MUT25	STANYNTSHLNNDVWQIFENPVDWKEK		
		MUT30	PSKPSFQEFVDWENVSPELNSTDQPFL		
		MUT33	DSGSPFPAAVILRDALHMARGLKYLHQ		
		MUT36	CGTAFFINFIAIYHHASRAIPFGTMVA		
		MUT44	EFKHIKAFDRTFANNPGPMVVFATPGM		
	universal TAAs	mSurvivin_20-28_	ATFKNWPFL	H-2D^b^	Lladser, (9)
		mSurvivin_53-67_	DLAQCFFCFKELEGW	I-A^b^	Ciesielski, (10)
		mTERT_198-205_	VGRNFTNL	H-2K^b^	Mennuni, (11)
**Balb/c mice bearing 4T1.2**	autologous tumour cell lysate^a^	Cell lysate-derived epitopes	N/A	N/A	Dolcetti^b^
	universal TAAs	mSurvivin_85-93_	AFLTVKKQM	H-2D^d^	Siegel, (12)
		mSurvivin_53-67_	DLAQCFFCFKELEGW	I-A^d^, I-E^d^	Results presented herein (**Figure S2**)
		mTERT1_67-175_	AYQVCGSPL	H-2K^d^	Mennuni, (11)

a Included in the vaccine formulation.

b Unpublished data.

TAAs: tumor associated antigens.

**Figure 1:**
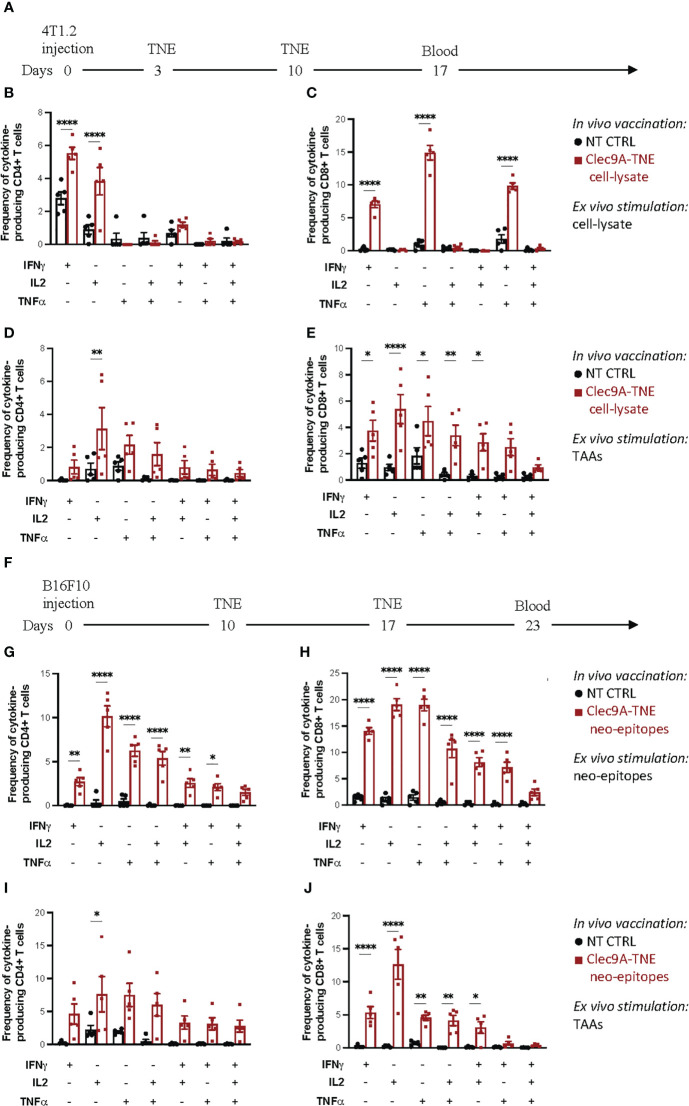
Polyfunctional assay on blood T cells. **(A)** Experimental scheme: Balb/c mice were orthotopically injected with 0.5×10^6^ 4T1.2 cells. Three and 10 days after tumor injection, mice were injected i.v. with Clec9a-TNE loaded with the 4T1.2 cell-lysate. On day 17 blood was collected and process as described in Methods. Cytokine production in CD4^+^ and CD8^+^ T cells was measured by flow cytometry and intracellular cytokine staining after *ex vivo* stimulation of blood samples with tumor lysate **(B, C)** or a pool of tumor associated antigen (TAA) for 6 h **(D, E)**. **(F)** Experimental scheme: C57BL/6 mice were subcutaneously injected with 0.25×10^6^ B16F10 cells. Ten and 17 days after tumor injection, mice were injected i.v. with Clec9a-TNE loaded with a pool of functionally validated, immunogenic B16F10 neo-epitopes. On day 23 blood was collected and process as described in Methods. Cytokine production in CD4^+^ and CD8^+^ T cells was measured by flow cytometry and intracellular cytokine staining after *ex vivo* stimulation of blood samples with B16F10 neo-epitopes **(G, H)** or a pool of tumor associated antigen (TAA) for 6 h **(I, J)**. Data are presented as mean values ± SEM. 0.1234 (ns), 0.0332 (*), (**) 0.0021, <0.0001 (****) (Two-Way ANOVA).

*Antibody staining for the detection of cytokine production.* At the end of the re-stimulation the cells were washed with 200 µL PBS by discarding the supernatant after centrifugation for 5 minutes at 500 rcf at 4°C. Pellets were resuspended in 100 µL of FVS440UV (BD Biosciences) working solution (1:1000 in PBS) and incubated for 15 minutes at room temperature in the dark. Cells were washed twice with 200 µL of MACS buffer (2mM EDTA pH8.0, 0.5% BSA in PBS). After the last wash, the supernatant was tipped off and the pellets were resuspended in the 50 µL of residual supernatant that remained. Non-specific antibody binding was limited by adding 0.5 µL of anti-mouse CD16/32 (BioLegend) to samples for 10 minutes on ice. An antibody master mix (**Table 2**) was then added for 30 minutes on ice, in the dark and the cells were then washed twice with 100 µL of MACS buffer. Samples were fixed using the eBiosciences FoxP3 kit according to manufacturer’s instructions. Samples were then left in 50 µL of the permeabilization buffer containing 0.5 µL of anti-mouse CD16/32 overnight at 4°C, protected from light. Incubation with intracellular antibodies was performed in a final volume of 100 µL of permeabilization buffer at room temperature, protected from light, for 1 hour. Samples were then washed with 100 µL of permeabilization buffer. After centrifugation for 5 minutes at 500 rcf at 4°C, samples were resuspended in 200 µL of MACS buffer for same-day acquisition on a BD FACSymphony A5 Cell Analyzer. Cytokine co-expression profiles were quantified using the Boolean function of Flowjo™ software. **Figure S1** describes the manual gating strategy carried out on blood cells (related to **Figure 1**)

**Table 2:** Flow cytometry antibodies used in the polyfunctional T cell assay.

	Specificity	Fluorochrome	Clone	Vendor	Dilution
**Surface staining**	CD3e	AF488	145-2C11	Biolegend	1/100
	CD8	APC/Cy7	53-6.7	Biolegend	1/100
	CD4	PE/Cy7	RM4-4	Biolegend	1/100
**Intracellular staining**	TNFα	AF647	MP6-XT22	Biolegend	1/100
	IFNγ	AF700	XMG1.2	Biolegend	1/100
	IL-2	PE	JES6-5H4	Biolegend	1/100

### 2.5 Staining Protocol for Multiparametric Flow Cytometry Panels 1 & 2

*Tissue processing*. Tissues were harvested immediately prior to processing. Spleens were manually dissociated and contaminating red blood cells were lysed with ACK buffer (150 mM NH4Cl, 10 mM KHCO3, 0.1 mM Na2EDTA). Peripheral lymph nodes and YUMM UV 1.7 tumors were manually dissociated. All samples were filtered with a 70 µM filter prior to processing for antibody staining. A viable cell count was performed, and 2 x 10^6^ cells were aliquoted into 1.5 mL polystyrene FACS tubes. Cells were washed with 600 µL PBS by discarding the supernatant after centrifugation for 5 minutes at 500 rcf at 4°C.

*Cell staining*. Cell pellets were resuspended in 100 µL of FVS440UV working solution (1:100 in PBS) and incubated for 15 minutes at room temperature in the dark. Cells were washed twice with 600 µL of MACS buffer (2mM EDTA pH8.0, 0.5% BSA in PBS). After the last wash, the supernatant was tipped off and the pellets were resuspended in the 50 µL of residual supernatant that remained. Non-specific antibody binding was limited by adding 0.5 µL of anti-mouse CD16/32 (BioLegend) to samples for 10 minutes on ice. An antibody master mix (**Table 3**) containing 10 µL of Brilliant Stain Buffer Plus (BD Biosciences) in a volume 50 µL, was then added to samples in 50 µL of MACS buffer; to make a final staining volume of 100 µL. Samples were stained for 30 minutes, on ice, in the dark and then washed with 1 mL of MACS buffer. Samples were fixed using the eBiosciences FoxP3 kit according to manufacturer’s instructions. Samples were then left in 100 µL of the permeabilization buffer overnight at 4°C, protected from light. Incubation with intracellular antibodies (**Table 3**) was performed at room temperature, protected from light, for 1 hour. Samples were then washed with 1 mL of permeabilization buffer. After centrifugation for 5 minutes at 500 rcf at 4°C, samples were resuspended in 200 µL of MACS for same-day acquisition on a BD FACSymphony A5 Cell Analyzer (**Tables 4**, **5**).

**Table 3:** List of antibodies and relative fluorochromes used in Panels 1 & 2 .

Panel	Specificity	Clone	Fluorochrome	Vendor	Titration	Purpose
**Shared Back bone**	CD45	30-F11	BUV563	BD – 565710	1/800	Lineage
	CD3	145-2C11	BUV737	BD – 564618	1/200	Lineage
	CD4	GK1.5	BUV496	BD – 564667	1/400	Lineage
	CD8	5.3-6.7	BUV805	BD – 564920	1/400	Lineage
	FoxP3*	FJK-16s	PE-Cy5	eBio – 15-5773-82	1/200	Lineage
	CD49b	HMα2	BV786	BD – OptiBuild	1/400	Lineage
	Amine-reactive	N/A	FVS440UV	BD – 566332	1/1000	Viability
	CD44	IM7	APC-Cy7	BD – 560568	1/400	Effector/memory
	CD279 (PD-1)	J43	BUV395	BD - OptiBuild	1/100	Inhibition
	Granzyme B*	QA16A02	PE-Dazzle594	BL – 372216	1/400	Activation
	CD69	H1.2F3	BV480	BD – OptiBuild	1/100	Activation
**Markers specific for Panel 1**	CD226 (DNAM-1)	TX42.1	BV650	BL – 133621	1/200	Activation
	CD314 (NKG2D)	CX5	BV711	BD – 563694	1/200	Activation
	CD152 (CTLA-4)	UC10-4F10-11	APC-R700	BD – 565778	1/200	Inhibition
	CD366 (TIM-3)	RMT3-23	FITC	eBio – 11-5870-82	1/400	Inhibition
	TIGIT	1G9	BV421	BD – 565270	1/200	Inhibition
	CD223 (LAG-3)	C9B7W	BV750	BD – OptiBuild	1/200	Inhibition
	VISTA	MIH64	PE	BD – 566270	1/200	Inhibition
	NKG2A/C/E	20d5	BV605	BD – 564382	1/200	Inhibition
	Eomes*	Dan11mag	PE-Cy7	eBio – 25-4875-82	1/200	Differentiation
	T-bet*	4B10	AF647	BL – 644804	1/400	Differentiation
**Markers specific for Panel 2**	CD62L	MEL-14	BB515	BD – 565261	1/400	Effector/memory
	CD103	2-E7	BV605	BL – 121433	1/100	Differentiation
	CD122	TM-β1	BB700	BD – *OptiBuild*	1/100	Differentiation
	CD127	SB/199	BV711	BD – 565490	1/100	Differentiation
	CD197 (CCR7)	4B12	BV650	BD – 564356	1/200	Differentiation
	CD25	PC61	APC-R700	BD – 565134	1/800	Differentiation
	Ki-67*	B56	PE	BD – 556027	1/400	Proliferation
	KLRG1	2F1	BV421	BD – 562897	1/200	Differentiation
	NKp46	29A1.4	PE-Cy7	BL – 137618	1/200	Differentiation
	TCF-1*	C63D9	AF647	CST – 6709S	1/200	Stemness

*Intracellular staining.

APC, Allophycocyanin; AF, Alexa Fluor; BUV, Brilliant Ultraviolet; BV, Brilliant Violet; PE, Phycoerythrin.

**Table 4:** Instrument Configuration for Panel 1.

Laser			Optics		Channels		Markers
Wavelength	Power	Type	LP Filter	BP Filter	Name	Flourochrome	Panel 1
**355 nm**	60 mW	DPSS	None	379/28	UV_379/28	BUV395	CD279 (PD-1)
			410	450/50	UV_450/50	FVS440UV	FVS440UV
			450	515/30	UV_515/30	BUV496	CD4
			550	586/15	UV_586/15	BUV563	CD45
			600	610/20	UV_610/20	−	
			635	670/30	UV_670/30	−	
			690	740/35	UV_740/35	BUV737	CD3
			770	820/60	UV_820/60	BUV805	CD8
**406 nm**	250 mW	DPSS	410	450/50	V_450/50	BV421	TIGIT
			505	525/50	V_525/50	BV480	CD69
			550	586/15	V_586/15	−	
			600	610/20	V_610/20	BV605	NKG2A/C/E
			635	670/30	V_670/30	BV650	CD226 (DNAM-1)
			690	710/50	V_710/50	BV711	CD314 (NKG2D)
			710	740/35	V_740/35	BV750	CD223 (LAG-3)
			750	780/60	V_780/60	BV786	CD49b
**488 nm**	150 mW	DPSS	505	515/20	B_515/20	FITC	CD366 (TIM-3)
			600	610/20	B_610/20	−	
			635	670/30	B_670/30	−	
			690	710/50	B_710/50	−	
			750	780/60	B_780/60	−	
			None	488/10	SSC	SSC	
			None	488/10	FSC	FSC	
**561 nm**	150 mW	DPSS	None	586/15	YG_586/15	PE	VISTA
			600	610/20	YG_610/20	PE-Dazzle594	Granzyme B
			635	670/30	YG_670/30	PE-Cy5	FoxP3
			690	710/50	YG_710/50	−	
			750	780/60	YG_780/60	PE-Cy7	omes
**637 nm**	140 mW	DPSS	None	670/30	R_670/30	AF647	T-bet
			690	710/50	R_710/50	APC-R700	CD152 (CTLA-4)
			750	780/60	R_780/60	APC-Cy7	CD44

APC, Allophycocyanin; AF, Alexa Fluor; BUV, Brilliant Ultraviolet; BV, Brilliant Violet; PE, Phycoerythrin.

**Table 5:** Instrument Configuration for Panel 2.

Laser			Optics		Channels		Markers
**Wavelength**	Power	Type	LP Filter	BP Filter	Name	Fluorchrome	Panel 2
**355 nm**	60 mW	DPSS	None	379/28	*UV_379/28*	BUV395	CD279 (PD-1)
			410	450/50	*UV_450/50*	FVS440UV	FVS440UV
			450	515/30	*UV_515/30*	BUV496	CD4
			550	586/15	*UV_586/15*	BUV563	CD45
			600	610/20	*UV_610/20*	−	
			635	670/30	*UV_670/30*	−	
			690	740/35	*UV_740/35*	BUV737	CD3
			770	820/60	*UV_820/60*	BUV805	CD8
**406 nm**	250 mW	DPSS	410	450/50	*V_450/50*	BV421	KLRG1
			505	525/50	*V_525/50*	BV480	CD69
			550	586/15	*V_586/15*	−	
			600	610/20	*V_610/20*	BV605	CD103
			635	670/30	*V_670/30*	BV650	CD197
			690	710/50	*V_710/50*	BV711	CD197 (CCR7)
			710	740/35	*V_740/35*	−	
			750	780/60	*V_780/60*	BV786	CD49b
**488 nm**	150 mW	DPSS	505	515/20	*B_515/20*	BB515	CD62L
			600	610/20	*B_610/20*	−	
			635	670/30	*B_670/30*	−	
			690	710/50	*B_710/50*	BB700	CD122
			750	780/60	*B_780/60*	−	
			None	488/10	*SSC*	SSC	
			None	488/10	*FSC*	FSC	
**561 nm**	150 mW	DPSS	None	586/15	*YG_586/15*	PE	Ki67
			600	610/20	*YG_610/20*	PE-Dazzle594	Granzyme B
			635	670/30	*YG_670/30*	PE-Cy5	FoxP3
			690	710/50	*YG_710/50*	−	
			750	780/60	*YG_780/60*	PE-Cy7	NKp46
**637 nm**	140 mW	DPSS	None	670/30	*R_670/30*	AF647	TCF-1
			690	710/50	*R_710/50*	APC-R700	CD25
			750	780/60	*R_780/60*	APC-Cy7	CD44

## 3 Results and discussion

### 3.1 Assay to characterize polyfunctional T cell responses to mouse tumor antigens

Cancer vaccine-induced antigen-specific T cell responses can be functionally characterized by *ex vivo* restimulation of peripheral blood cells, splenocytes or isolated tumor infiltrating lymphocytes with the antigens targeted by the vaccine in the form of peptide epitopes or, less frequently, tumor cell lysates. The expression of IFNγ, TNFα and IL-2 by intracellular staining and flow cytometry analysis can be used as read out where polyfunctionality of antigen-specific CD4^+^ and CD8^+^ T cell responses is given by the extent of stimulated T cells expressing more than one cytokine. Additional functional markers can be also investigated such as CD137, CD107, as well as cytolytic molecules (perforin and granzyme B). Tumor cell lysates can be used for restimulation when no suitable tumor antigen is available. **Figure 1A-E** shows the results of a polyfunctional T cell assay carried out on blood cells isolated from Balb/c mice carrying a 4T1.2 triple negative breast cancer and vaccinated with Clec9a-TNE loaded with the 4T1.2 cell-lysate. Blood cells from controls and vaccinated mice were *ex vivo* restimulated with the tumor cell lysate used for the vaccination (**Table 1**). As presented in **Figure 1C**, vaccinated mice showed significantly higher percentages of CD8^+^ T cells expressing IFNγ, TNFα or both cytokines as evidence of polyfunctionality when compared with control mice. In vaccinated mice, the assay also revealed higher percentages of CD4^+^ T cells expressing IFNγ or IL-2 as single cytokines, whereas only a slight increase in CD4^+^ T cells expressing both cytokines was observed (**Figure 1B**). This data indicate that the lysate included in the vaccine generated only partial tumor-specific polyfunctional T cell responses, mainly involving CD8^+^ T cells. These results are consistent with the limited immunogenicity of tumor cell lysates, which need to be *ex vivo* manipulated to enhance their therapeutic efficacy (1).

The recent advances in genomics, trancriptomics and tumor epitope prediction allowed the use of neo-antigen epitopes identified in individual tumors to track and characterize neo-antigen specific T cell responses in cancer patients treated with personalized vaccines (1, 13, 14). Tumor neo-epitopes may be also available for some preclinical cancer models (14), allowing thus a more precise assessment of the quality of tumour-specific T cell responses induced in these models by a variety of experimental immunotherapeutic strategies. **Figures 1G, H** shows the results of polyfunctional responses of CD4^+^ and CD8^+^ T cells restimulated with the highly immunogenic B16F10 neo-epitopes (8) used to vaccinate tumor-bearing C57BL/6 mice (**Figure 1F**).

Although feasible (**Figures 1B, C**), the characterization of tumor-specific polyfunctional T cell responses in mice vaccinated with tumor cell lysates is hampered by the lack of knowledge of the immunogenic (neo-)antigens targeted by the vaccine. Although re-stimulation of T cells with the whole tumor cell lysate can be an option, this approach suffers from variability among cell lysate batches that may lead to inconsistent results. To obviate these limitations, we exploited epitopes of the “universal” TAAs telomerase and survivin, for which CD4 and CD8 immunogenic epitopes are available for both Balb/c and C57BL/6 mouse strains (**Table 1**). To complete the panel of suitable “universal” TAAs applicable in the most common preclinical models, we have validated the immunogenicity of the mSurvivin_53-67_ epitope so far applied in C57BL/6 cancer models also in the Balb/c mouse strain (**Figure S2**). **Figures 1D, E** reports the results of a polyfunctional T cell assay carried out on blood cells isolated from 4T1.2-bearing Balb/c mice vaccinated with an autologous tumour cell lysate, and *ex vivo* re-stimulated with telomerase and survivin epitopes. Higher percentages of CD4^+^ T cells expressing IL-2 and CD8^+^ T cells expressing IFNγ, IL-2, TNFα or the IFNγ/IL-2, and TNFα/IL-2 combinations (polyfunctionality) were observed in immunized mice. These epitopes can be also useful to identify and characterize epitope spreading in both C57BL/6 and Balb/c mice vaccinated with different antigenic formulations or treated with other immunotherapeutic strategies. As representative example of this application, **Figures 1I, J** reports the CD4^+^ and CD8^+^ T cell responses specific for telomerase and survivin detected in C57BL/6 mice bearing B16F10 melanoma and vaccinated with autologous tumor neo-epitopes.

### 3.2 Integrated multiparametric flow cytometry assays to immune profile mouse T and NK cells.

**Figure 2:**
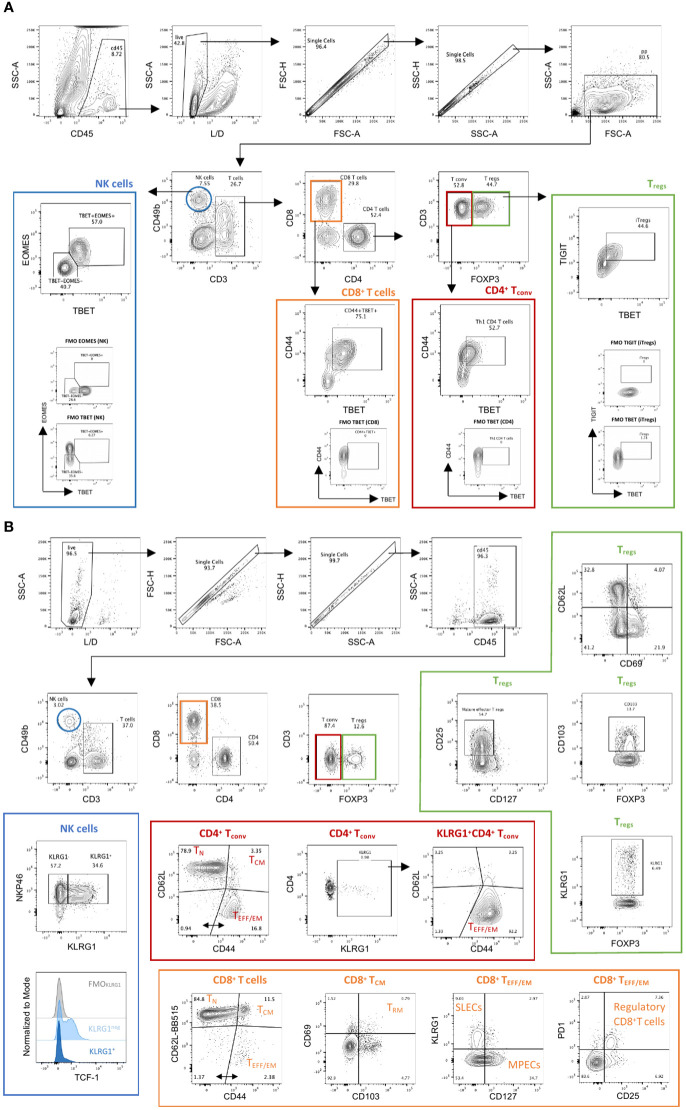
Manual gating strategy in Panels 1 & 2. **(A)** Panel 1. Tumor infiltrating immune cells from YUMM UV 1.7 tumors were identified through exclusion of non-viable and CD45^−^ cells, followed by use of physical parameters to distinguish lymphocytes and exclude doublets. Main immune cell subsets were defined as follows: natural killer cells (blue; NK; CD3^−^CD49b^+^); conventional CD4^+^ T cells (red; CD4^+^ Tconv; CD3^+^CD4^+^FoxP3^−^); regulatory CD4^+^ T cells (green; Treg; CD3^+^CD4^+^FoxP3^+^) and CD8^+^ T cells (orange, CD3^+^CD8^+^). Examples of additional subpopulations are also indicated as follow: differentiated and cytotoxic NK cells (T-bet^+^Eomes^+^); Th1 CD4^+^ T cells (CD4^+^CD44^+^T-bet^+^); induced Tregs (iTregs: CD4^+^FoxP3^+^T-bet^+^TIGIT^+^); Effector CD8^+^ T cells (CD8^+^CD44^+^T-bet^+^). Fluorescent minus one (FMOs) for Eomes, T-bet and TIGIT are also shown. **(B)** Panel 2. Spleens isolated from C57Bl/6 mice bearing YUMM UV 1.7 melanomas were processed as described in methods. Immune cells were identified through exclusion of non-viable and CD45^−^ cells; followed by use of physical parameters to distinguish lymphocytes and exclude doublets. Main immune cell subsets were defined as in **(A)**. Examples of additional subpopulations are also indicated as follow: mature and differentiated NK cells (NKp46^+^KLRG1^+^); Naïve (T_N_: CD62L^+^CD44^-^), central memory (T_CM_: D62L^+^CD44^+^) or effector/effector memory (T_EFF/EM_: CD62L^-^CD44^+^) CD4^+^ and CD8^+^ T cells; Cytotoxic CD4^+^ T cells (CD4^+^FOXP3^-^KLRG1^+^); Tissue resident memory CD8^+^ T cells (T_RM:_ CD69^+^CD103^+^CD8^+^ T_CM_); short lived effector cells (SLECs: KLRG1^+^CD127^-^ CD8^+^ T_EEF/EM_) and memory precursor cells (MPECs: KLRG1^-^CD127^+^ CD8^+^ T_EEF/EM_); regulatory CD8 T cells (PD1^+^CD25^+^CD8^+^ T_EEF/EM_).

Two flow cytometry panels were designed to simultaneously allow the comprehensive characterization of NK and T cell exhaustion, activation, functionality, transcription factor profile, memory and differentiation states. When used in combination, these panels can provide a comprehensive overview of the functional status of T and NK cells in the tumor microenvironment and peripheral lymphoid organs in mice. Moreover, the two panels can also be used separately for focused investigation of activation/exhaustion (Panel 1) or memory phenotypes (Panel 2) in a variety of disease settings. The panels share a set of common hematopoietic and lineage markers as backbone (**Table 3**). In both panels, CD45 was included to identify hematopoietic cells in complex tissues, and this can be adapted to track transferred T cells based on their allotype (CD45.1/CD45.2) in adoptive cell transfer studies. Briefly, in both panels, CD45 and a live/dead marker are used to first gate live hematopoietic cells. FSC and SSC are then used to gate lymphocytes followed by doublet discrimination. Major T cell subsets are identified using CD3, CD4, CD8 and FoxP3, whereas NK cells are identified with CD49b (**Figure 2** & **Figure S3**). The results presented were obtained in the YUMM 1.7 UV mouse melanoma model, which closely recapitulates the main genetic and immunologic features of human cutaneous melanoma (7).

### 3.3 Panel 1: Activation and exhaustion phenotypes

**Figure 3:**
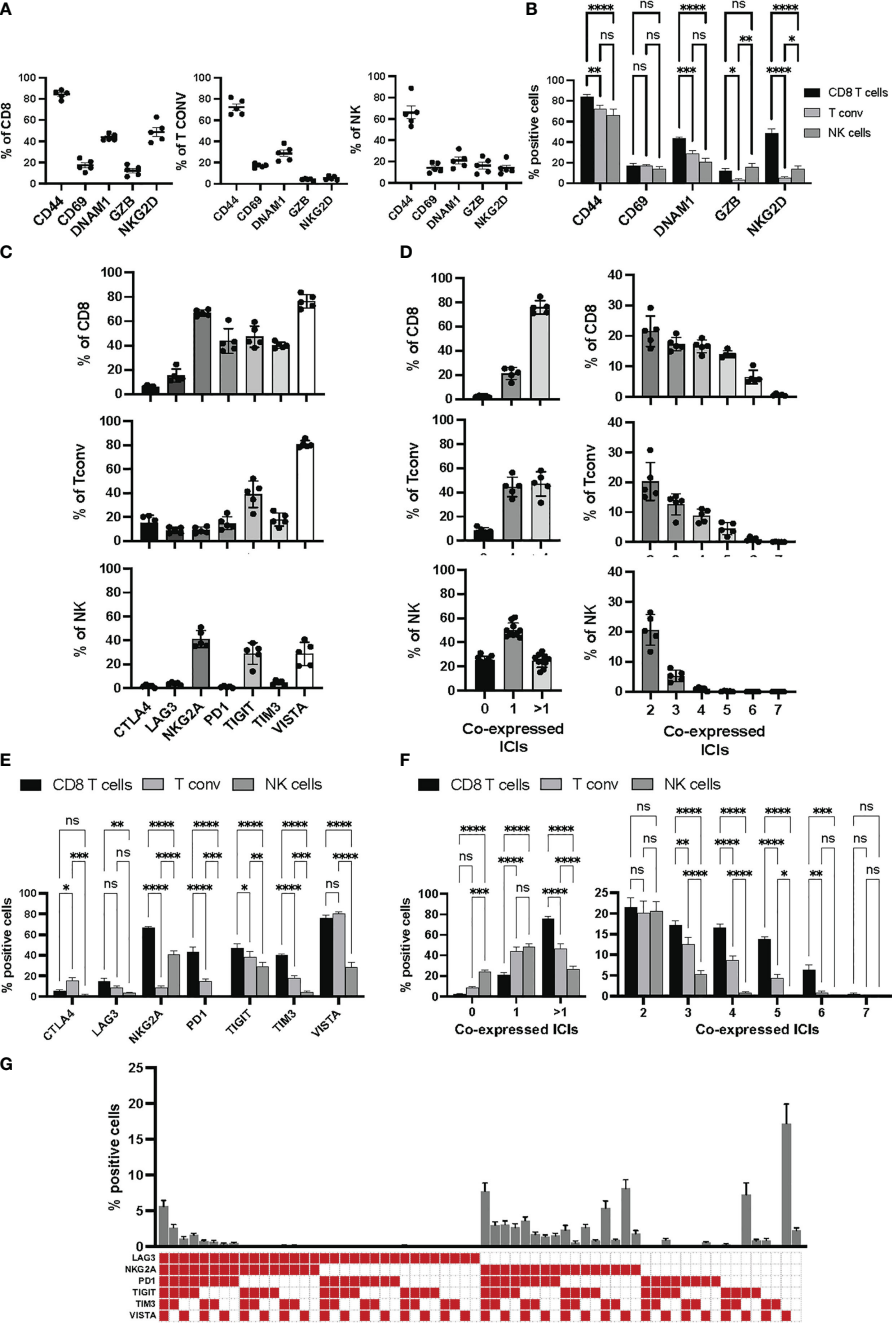
Activation and immune checkpoint marker expression in Panel 1. Tumor infiltrating immune cells from YUMM UV 1.7 tumors were analyzed as described in **Figure 2A**. **(A)** Percentages of cells expressing the indicated activation markers within the CD8^+^ (left), T_conv_ (middle) or NK (right) cell populations. **(B)** Percentages in **(A)** were grouped to directly compare differences in the percentages of each activation markers across CD8^+^ T cells, T conv, and NK cells. Data are presented as mean values ± SEM. 0.1234 (ns), 0.0332 (*), 0.0021 (**), 0.0002 (***), <0.0001 (****) (Two-Way ANOVA, Fisher’s LSD test). **(C)** Percentages of cells expressing the indicated immune checkpoint molecules within the CD8^+^ (top), T_conv_ (middle) or NK (bottom cell populations. **(D)** Boolean gating for the identification of the percentage of cells expressing 0, 1 or >1 immune checkpoint inhibitors (ICIs, left column) and 2 to 7 immune checkpoint inhibitors (ICIs, right column) within the CD8^+^ (top), T_conv_ (middle) or NK (bottom) cell populations. **(E)** Percentages in **(C)** were grouped to directly compare differences in the percentages of each immune checkpoint inhibitor across CD8^+^ T cells, T conv, and NK cells. Data are presented as mean values ± SEM. 0.1234 (ns), 0.0332 (*), 0.0021 (**), 0.0002 (***), <0.0001 (****) (Two-Way ANOVA, Fisher’s LSD test). **(F)** Percentages in **(D)** were grouped to directly compare differences in the percentages of cells co-expressing the indicated numbers of immune checkpoint inhibitors (ICIs) across CD8^+^ T cells, T_conv_, and NK cells. Data are presented as mean values ± SEM. 0.1234 (ns), 0.0332 (*), 0.0021 (**), 0.0002 (***), <0.0001 (****) (Two-Way ANOVA, Fisher’s LSD test). **(G)** Boolean gating (FLowJo) for 6 different immune checkpoint inhibitors on tumor infiltrating CD8^+^ T cells. The graph represents the percentage of tumor infiltrating CD8^+^ T cell positive for any of the immune checkpoint combinations indicated by the red square below the x axes. Data are presented as mean values ± SEMs, n=5.

In addition to the backbone lineage markers, to address activation vs. exhaustion in T and NK cells, the evaluation of the transcription factors Eomesodermin (Eomes) and T-bet was incorporated in Panel 1 given their role in T and NK cell differentiation, activation and exhaustion (15–17) (**Figure 2A**). To specifically assess activation, antigen experience, and cytolytic activity, CD44, CD69, NKG2D, DNAM-1 and Granzyme-B were included in Panel 1 (**Figures 3A, B** & **Figure S4**). Finally, a suite of inhibitory immune checkpoints (TIGIT, VISTA, TIM-3, CTLA-4, NKG2A, PD-1, LAG-3) were added to evaluate the exhaustion state of the different subpopulations during tumor progression and following treatments (**Figures 3C, E** & **Figure S4**)

#### 3.3.1 NK cells

Within NK cells (**Figure 2A**, blue frame), the inhibitory receptors NKG2A, TIM-3, PD-1, LAG-3 and TIGIT together with the activating receptor NKG2D and DNAM-1 can be used to evaluate the effector function of CD3^-^CD49b^+^ NK cells. The balance between activating and inhibitory receptors determines whether NK cells will have cytolytic functions or contribute to a tolerogenic microenvironment (18). To investigate whether impaired effector functions are associated with reduced activation or cytolytic activity, the early activation and immune modulator CD69, as well as the cytolytic molecule Granzyme-B, can be assessed. The dysfunctional and exhausted phenotype of tumor-associated NK cells can additionally be investigated by analyzing the expression of Eomes and T-bet, T‐box transcription factors that drive the differentiation and function of cytotoxic lymphocytes and NK cells (15). In the context of tumor progression, upon adoptive transfer to tumor‐bearing mice, NK cells were shown to display a progressive decrease in the expression of both transcription factors and this correlated with a rapid loss of effector function including the ability to control metastasis development (19).

#### 3.3.2 CD4^+^ T cells

Within CD4^+^ T cells, the two main regulatory T cell subsets, Tregs (**Figure 2A**, green frame) and type 1 regulatory T cells (TR1), can be identified based on FoxP3 and Eomes expression, respectively (19, 20) and distinguished from conventional CD4^+^ T cells (**Figure 2A**, red frame). CD44, a marker commonly used to assess activation in T cells, can also be employed to identify antigen-experienced T cells. CD44 expression is rapidly up-regulated after antigen encounter and is maintained in memory T cells. T-bet and Eomes were included to report on their expression profile in various CD4^+^ T-helper subsets. Eomes plays an important role in cytotoxic T lymphocytes where it promotes their development and survival *via* upregulation of CD122 (the β-chain of the IL‐15 receptor) and represses Th17‐type cytokine expression (15). Expression of T‐bet and Eomes is induced upon activation and differentiation. Moreover, T‐bet promotes Th1 cell differentiation and prevents the development of Th2 and Th17 helper cells. Recent studies have reported that both circulating and tumour-infiltrating Th1-like CD4^+^ T cells expressing T-bet display strong anti-tumour cytotoxic activities (21) (**Figure 2A**, red frame). However, TGFß-mediated conversion of anti-tumor T-bet^+^Th1 CD4^+^ T cells into immunosuppressive T-bet^+^Foxp3^+^ Tregs was recently described in mice bearing lung carcinoma (22). TIGIT, a co-inhibitory receptor, is highly expressed on Tregs that specifically inhibits Th1 and Th17 responses (23–25), also known as induced T_regs_ (iT_regs_: Tbet^+^TIGIT^+^, **Figure 2A**, green frame).

#### 3.3.3 CD8^+^ T cells

CD44, CD69 and Granzyme-B were included as classical markers to evaluate activation and cytolytic activity of CD8+ T cells (**Figure 2A**, frame orange), with DNAM-1 and NKG2D included to provide further information on the activation status of the CD8^+^ T cell compartment. Additionally, NKG2D can work as a co-stimulatory molecule in antigen-experienced T cells (26). DNAM-1 acts as a co-stimulatory factor in CD8^+^ cytotoxic T lymphocytes and is down-regulated in dysfunctional tumor-infiltrating CD8^+^ T cells (27, 28). The two transcription factors T-bet and Eomes were used to further evaluate the differentiation status of CD8^+^ T cells. Indeed, high T-bet expression has been associated with an effector phenotype, and high expression of Eomes with a memory phenotype (29, 30). Finally, various immune checkpoints (TIGIT, VISTA, TIM-3, CTLA-4, NKG2A, PD-1, LAG-3) were included to delineate activation vs. exhaustion, and to aid in the identification of dysfunctional CD8^+^ T cells in the tumor microenvironment. Of note, the co-expression of immune checkpoints with DNAM-1 on CD8^+^ T cells, particularly in hematological malignancies, is indicative of activation rather than exhaustion (28, 30). DNAM-1 and TIGIT share the same ligands (CD155 and CD112), however, they have opposing functions (activation vs. suppression, respectively) in tumor infiltrating CD8^+^ T cells (27).

#### 3.3.4 T cell exhaustion signatures

T cell exhaustion signatures may vary between different tumor types. However, they are almost invariably associated with co-expression of several classical immune checkpoint molecules such as PD-1, CTLA-4, TIM-3, LAG-3 and TIGIT. Indeed, high PD-1 expression levels on effector memory T cells can signal either activation or exhaustion depending on the level and repertoire of additional checkpoint molecules expressed (31). It is the progressive upregulation of these additional inhibitors, and loss of co-stimulatory molecules, that determines the hierarchical loss of effector functions and acquisition of dysfunctional/exhausted phenotypes (**Figure 3C-G**).

#### 3.3.5 LAG-3

LAG-3 is upregulated on activated CD4^+^ and CD8^+^ T cells, as well as on a subset of NK cells. Importantly, LAG-3 and PD-1 are frequently co-expressed on both CD4^+^ and CD8^+^ tumor infiltrating lymphocytes (TILs) and their co-blockade has been shown to synergize to improve anti-tumor responses in several pre-clinical murine cancer models (32). LAG-3 is highly expressed in Treg cells and its blockade abrogates Treg cell suppressor functions (33). LAG-3 is also expressed on type 1 regulatory (Tr1) T cells, identified in both humans and mice as FoxP3^neg^LAG-3^+^CD49b^+^ CD4^+^ T cells (32).

#### 3.3.6 TIM-3

TIM-3 is a negative regulator of type 1 immunity expressed on IFN-γ-producing CD4^+^ Th1 helper cells, CD8^+^ cytotoxic T cells, T_regs_ cells as well as on innate immune cells (dendritic cells, NK cells, monocytes). In these cell types, TIM-3 inhibits immune responses and promotes tolerance (34). Tumor infiltrating CD8^+^ T cells co-expressing TIM-3 and PD-1 can exhibit a severely dysfunctional or exhausted phenotype and TIM-3 and PD-1 co-blockade is more effective than PD-1 blockade alone in improving anti-tumor immune effector functions in preclinical models of both solid and hematologic cancer (32). TIM-3 also regulates the function of FoxP3^+^ T_reg_ cells and TIM-3^+^ T_regs_ display higher expression of known T_reg_ effector molecules such as IL-10, Granzymes, and perforin. In the context of cancer, high level of TIM-3 on NK cells is associated with a dysfunctional and exhausted phenotype and TIM-3 blockade restores NK cell functions (32).

#### 3.3.7 TIGIT

TIGIT is highly expressed on human and murine TILs in several tumor types. It is often co-expressed with PD-1, TIM-3, and LAG-3 whose co-expression in the absence of co-stimulatory molecules can identify the most dysfunctional CD8^+^ TILs (24, 27). TIGIT ligands CD155 and CD112 are widely expressed on tumor cells. DNAM-1 (CD226), the agonistic signal of this co-stimulatory pathway, promotes cytotoxicity and enhances anti-tumor responses. In contrast, TIGIT negatively regulates anti-tumor responses, but with a specialized role in the tumor tissue as it is not expressed in peripheral lymphoid organs (34). Of note, TIGIT also promotes the immunosuppressive activity of tumor tissue Treg cells (34, 35). Therefore, targeting TIGIT with monoclonal antibodies may not only enhance effector T cell function, but also suppresses TIGIT cell-mediated immunosuppression (27, 36).

#### 3.3.8 VISTA

VISTA is predominantly expressed in hematopoietic cells with the highest level of expression observed in myeloid cells, particularly microglia and neutrophils, followed by monocytes, macrophages, and dendritic cells. VISTA is also highly expressed on naïve CD4^+^ and CD8^+^ T cells, as well as Foxp3^+^ Treg cells. While most immune checkpoint modulators fine-tune T cell response and fate after activation, VISTA was recently shown to regulate naïve T cell quiescence and peripheral T cell tolerance (37). VISTA is also emerging as a novel targetable immune checkpoint in oncology and its altered expression has been prognostically implicated in different types of cancer (38).

#### 3.3.9 NKG2A

NKG2A is an inhibitory member of the NKG2 family, it dimerizes with CD94 on the cell surface and its major ligand is the non-classical MHC class I molecule HLA-E. NKG2A is expressed on NK cells, natural killer T (NKT) cells and a subset of CD8^+^ T cells. Importantly, exhausted TILs can display high NKG2A expression, and this correlates with reduced survival in patients with ovarian or colorectal cancer (39).

### 3.4 Panel 2: Memory phenotypes

The ability to preclinically evaluate the capacity of a new treatment, or a novel combination therapy, to enhance anti-tumor immune responses and subsequent formation of memory populations is vital to formulating new treatment strategies to translate into the clinic. To this end, we have designed and optimized a multiparametric flow cytometry panel (Panel 2) to specifically assess changes in memory cell differentiation, proliferation and effector functions in mouse immune cells (**Table 3**). The same backbone designed for Panel 1 to identify major T and NK cell subset was used for this panel thus allowing a broad applicability and the simultaneous investigation of activation/exhaustion and memory phenotypes (**Figure 2B** & **S3**).

#### 3.4.1 NK cells

To identify mature, effector NK cells, CD49b^+^ cells were further characterized for the co-expression of NKp46, which is used in mice to specifically and accurately identify NK cells (40), and KLRG1 (**Figure 2B** and **Figure S3**, blue frame). Consistently, NKp46^+^KLRG1^+^ cells lack TCF-1 expression (41). However, because KLRG1 may have inhibitory functions upon binding to cadherins abundantly expressed in some tumor microenvironments, its monitoring may inform on the immune modulatory activity of a given therapy on NK cells in a context-dependent manner (42). Of note, the addition of CD25 in this panel also allows the monitoring of “memory-like” NK cells, a subpopulation with enhanced recall function to multiple stimuli (43–45).

#### 3.4.2 T cells

For T cells, CD44 and CD62L gating was used to identify naïve (T_N_), central memory (T_CM_) and effector/effector memory (T_EFF/EM_) subsets (**Figure 2B**; **Figure S3**). A marker for ‘stemness’, TCF-1, was included as it has recently been described as an important indicator of T cell self-renewal capability and, when co-expressed with PD-1, as marker of responsiveness to immune checkpoint inhibition (46, 47). The expression of Ki67 allows the identification of proliferating T cells providing a potential means to identify T cell populations that are responding to any immunotherapy or vaccination (48). CCR7 was included in this panel to assess T cell trafficking, particularly in lymph node samples, and to monitor T cell differentiation (49).

#### 3.4.3 CD8^+^ T cells

Within the CD8+ TCM subset (**Figure 2B** and **Figure S3**, orange frame), CD122 expression has been identified as a marker denoting antigen experience (50), whereas the expression of CD69 and/or CD103 may identify tissue-resident memory T cells (51, 52). Within the T_EFF/EM_ subset, KLRG1 and CD127 are utilized to delineate short lived effector cells (SLECs) and memory precursor cells (MPECs) (53). Moreover, within the same subset, assessment of PD-1 and CD25 expression allows the identification of suppressive and/or regulatory CD8^+^ T cells (54, 55). In tumor draining lymph nodes, co-expression of PD-1 and TCF1 can identify precursors of exhausted T cells (T_PEX_), which are known for their abilities to self-renew and for their properties of functionally restrained effector cells that are emerging as important mediators of cancer immunotherapy (21, 47).

#### 3.4.4 CD4^+^ conventional T cells

Conventional CD4+ T cells (**Figure 2B** and **Figure S3**, red frame) with direct cytotoxic activity are emerging as important players in anti-tumor immunity (56). In this panel, focusing on CD4^+^FoxP3^-^ T cells, KLRG1 can be used to identify cytotoxic CD4^+^ T cells, which display an effector phenotype (57, 58).

#### 3.4.5 Tregs

Within CD4+FOXP3+ T_regs_ (**Figure 2B** and **Figure S3**, green frame), CD25 is a useful marker for assessing maturation of these cells (59). CD69 can additionally be used to identify a Treg cell subset known to express high levels of IL-10 in a STAT3-dependent manner (60). CD103 can be used to identify another T_regs_ cell subset present in both lymphoid tissues and tumors, whose expression levels correlate with the level of TGFß secreted by tumor cells (61, 62). KLRG1 was applied to identify a small proportion of T_regs_ in the periphery that exhibit a gene expression profile of “activated and terminally differentiated effector T_regs_ (63).

### 3.5 Panel 1 & 2: data analysis

**Figure 4:**
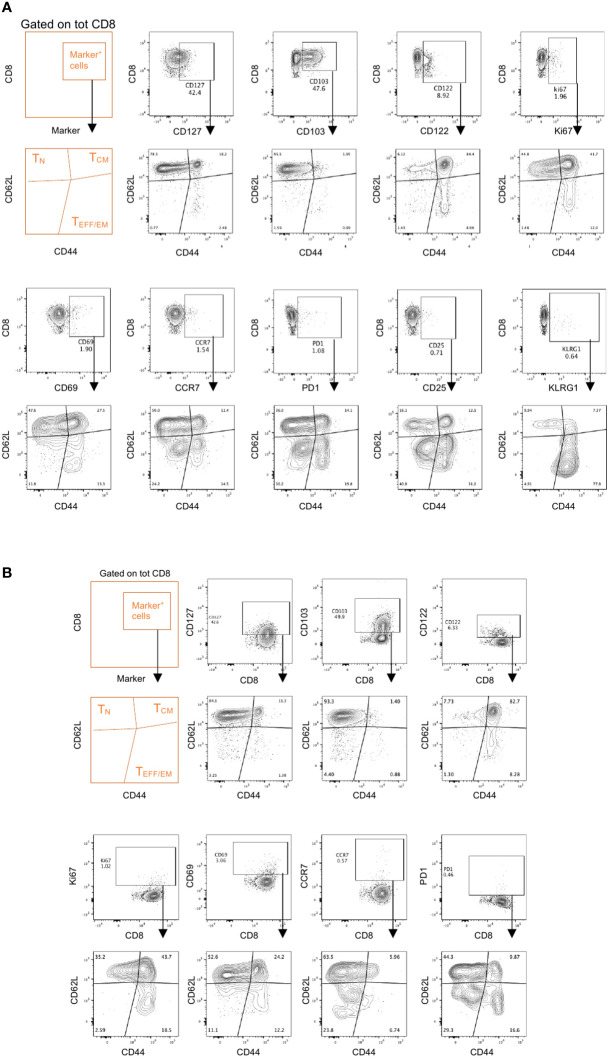
Memory effector phenotypes in Panel 2. Spleens and draining lymph nodes isolated from C57Bl/6 mice bearing YUMM UV 1.7 melanomas were processed and stained with Panel 2 as described in methods. **(A, B)** Representative analysis on CD8^+^ T cells from spleens **(A)** or tumor draining lymph nodes **(B)** where marker positive cells (top contour plots) are interrogated for their memory/effector phenotype (bottom contour plots) using the CD62L and CD44 markers.

**Figure 5:**
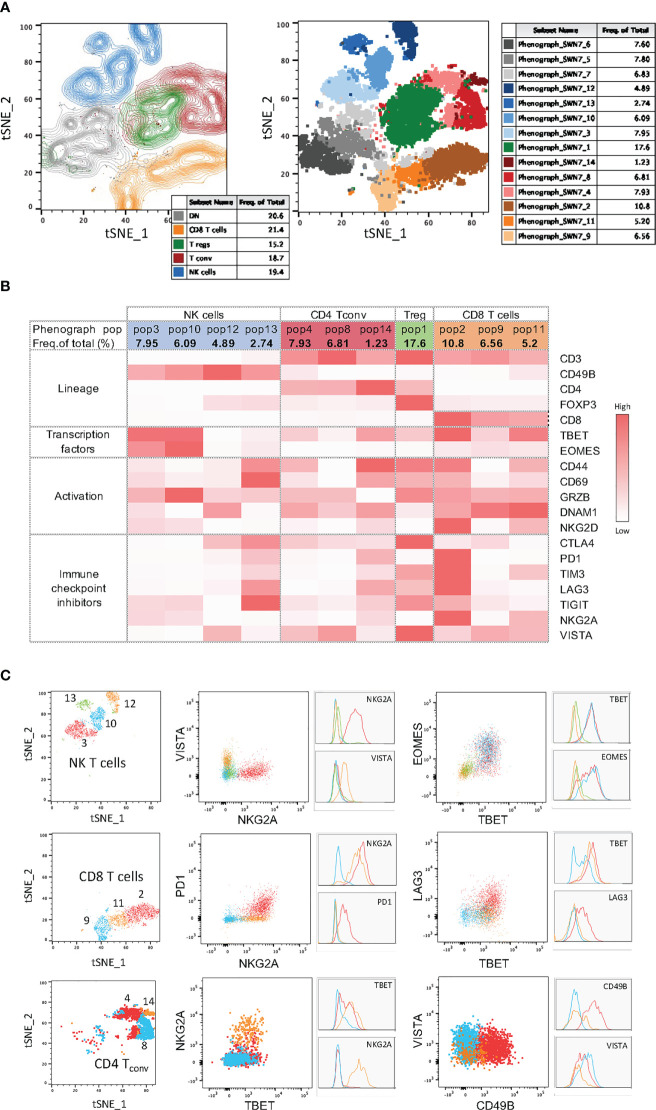
High-dimensional data analysis on tumor infiltrating immune cells from YUMM UV 1.7 tumors stained with Panel 1. **(A)** Left: tSNE plots on concatenated T and NK cells from 4 YUMM UV 1.7 tumors with overlayed colored manual gates for CD8^+^ (orange), CD4^+^ T_conv_ (red), T_regs_ (green), NK (blue), and double negative (CD4^-^CD8^-^) T cells (grey). Contour plots reveal cell density and main subpopulations. Right: Same tSNE as on the left where cells are color-coded according to the 14 clusters identified with PhenoGraph. **(B)** Mean fluorescence intensity (MFI) heatmap for the indicated markers across the 4 main immune cell population and within each cluster obtained with PhenoGraph. Marker expression intensity is indicated by the scale bar at the right of the table where white is low and red is high. **(C)** Left column: tSNE plots showing cluster overlays in 3 main immune cell population: NK cells, CD8^+^ T cells, and CD4^+^ T_conv_. Middle and right colums: same cluster overlays analysed for differentiation and exhaustion markers. Both dot plots and corresponding histograms are presented.

**Figure 6:**
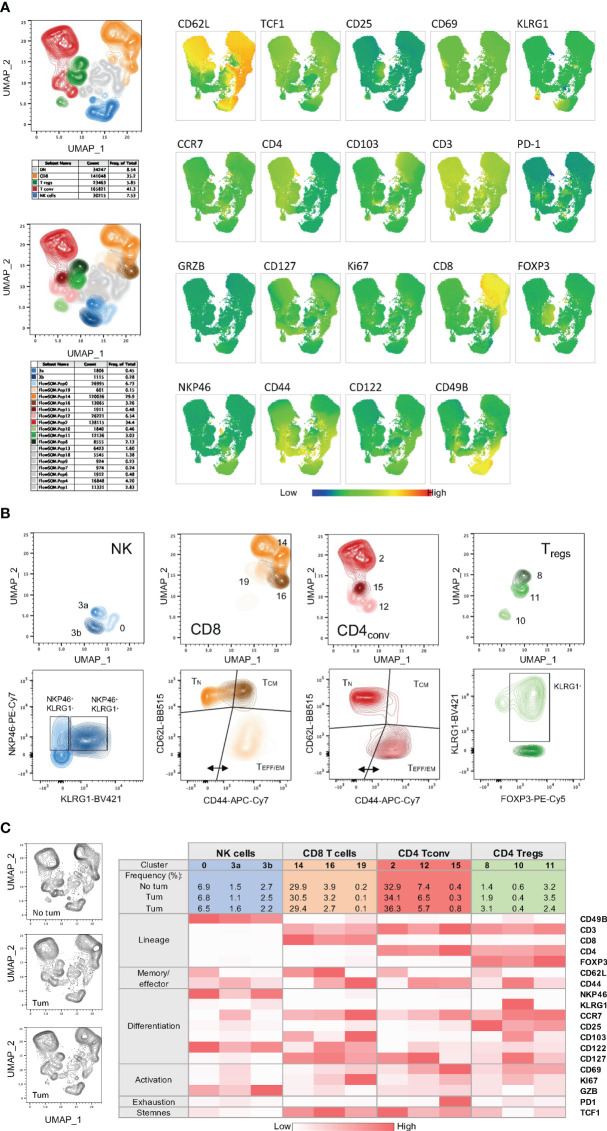
High-dimensional data analysis on splenocytes from mice bearing YUMM UV 1.7 tumors and stained with Panel 2. **(A)** Top left: UMAP plot on concatenated T and NK cells from 3 spleens isolated from C57Bl/6 mice bearing YUMM UV 1.7 tumours. The plot shows overlayed coloured manual gates for CD8^+^ T cells (orange), CD4^+^ T_conv_ cells (red), CD4^+^ T_reg_ cells (green), NK cells (blue), and double negative (CD4^-^CD8^-^) T cells (grey). Contour plots reveal cell density and main subpopulations. Bottom left: Same UMAP as in the top plot where cells are color-coded according to the 17 main clusters identified with FlowSOM and additional sub-clusters (3a and 3b) manually defined. Right: visualization of the expression phenotype across all T and NK cell clusters in UMAP. Marker expression intensity is indicated by the scale bar at the bottom of the plots where blue is low and red is high. Each marker is indicated above each plot. **(B)** Top plots: UMAP plots showing clusters and sub-clusters in the 4 main immune cell population: CD8^+^, CD4^+^ T_conv_, CD4^+^ T_regs_ and NK cells. Bottom plots: same clusters and sub-clusters analysed for maturation and differentiation markers. **(C)** Left: UMAP plots from individual spleens. Right: MFI heatmap for the indicated markers across the 4 main immune cell population and within each cluster and sub-cluster obtained with FlowSOM or manually defined. Marker expression intensity is indicated by the scale bar at the bottom of the table where white is low and red is high.

The main purpose of these two integrated flow cytometry panels is to allow the simultaneous analysis of the co-expression of multiple checkpoint inhibitors and the characterization of the memory/effector status of distinct T cell and NK cell subpopulations. With regard to Panel 1, this can be achieved by combining manual gating of known sub-populations (**Figures 2A**, **3A, B, C, E**) with Boolean gating (**Figures 3D, F, G**). This approach allows the assessment of the proportions of cells expressing none, one or different combinations of multiple immune checkpoint molecules within a specific (known) subpopulation of interest. Back-gating on the parental population against specific activation/differentiation markers of interest provides additional information on the functional status of the immune checkpoint expressing cells (**Figure S4**). For Panel 2, manual gating of known sub-populations can be used followed by downstream analysis within a specific subset (naïve versus effector/memory) for the characterization of additional subpopulations of functional relevance (**Figure 2B**; **Figure S3**). Alternatively, the differentiation status (naïve versus effector/memory) of immune cell subsets, such as CD8^+^ T cells (**Figure 4**), expressing specific combinations of markers can be elucidated using this panel. Moreover, for an unbiased interrogation of the data, dimension reduction techniques such as tSNE or UMAP combined with clustering algorithms (FlowSOM and PhenoGraph) can be used to identify known and novel subpopulations characterized by the co-expression of specific differentiation, activation, and/or checkpoint molecules in both Panels (**Figures 5**, **6**; **S5**).

During the development of these integrated panels, antigen classification, resolution ranking, and an instrument specific spillover spread matrix (**Figure S6** and **Tables S1**, **S2**) were used to predict the best fluorochrome combination and fluorochrome/antigen association listed in **Table 3**. Please refer to online supplemental material for the optimization of the panel, antibody titration and detailed technical information (**Figures S6–S11**).

## 4 Conclusive remarks

One limitation of the results presented herein is the application of the polyfunctional T cell assays to blood immune cells. Although this approach can allow a reliable monitoring of systemic tumor-specific T cell responses over time during immunotherapeutic treatments, it may not accurately reflect the real situation in the tumour microenvironment. However, the same protocol can easily applied to *ex vivo* cultured tumour infiltrating lymphocytes provided that adequate numbers of these cells can be obtained from tumor tissues. Similar approaches assessing the extent of polyfunctional T cell responses to tumor antigens have been successfully applied to the peripheral blood immune cells of tumor patients, providing useful indication of changes in adaptive immunity responses induced by treatment and allowing the identification of potential immune biomarkers predictive of the response to therapy (64, 65). Moreover, the multicolor flow cytometry panels we developed for mouse cells can be easily adapted to characterize T cell/NK cell exhaustion and memory phenotypes of human immune cells. These panels may be particularly useful to identify changes in the expression of targetable immune checkpoint molecules thus allowing a rational tailoring of combination immunotherapy for cancer patients. These panels, however, should take into account the differences between mouse and humans with regard to the level of expression and biological meaning of distinct immune markers.

In summary, the integrated exploitation of the multiparametric flow cytometry-based methods we developed can allow a reliable preclinical assessment of vaccine-induced polyfunctional T cell responses, also including the epitope spreading, and the concomitant characterization and T cell/NK cell exhaustion and memory phenotypes in mouse immuno-oncology models. Application of these protocols may improve the definition of the most effective antigen formulations for next generation cancer vaccines and allow the identification of novel mechanism-based combination immunotherapies.

## Data availability statement

The raw data supporting the conclusions of this article will be made available by the authors, without undue reservation.

## Ethics statement

The animal study was reviewed and approved by University of Queensland Animal Ethics Committee (approval number UQDI/252/16).

## Author contributions

RD, SM, DS and RM designed the research. DM, BZ, SM, RB and JW performed the experiments. DM, BZ, SM, RM and RD analyzed the data. SM and RM drafted the manuscript. RM and RD revised the manuscript. All authors contributed to the article and approved the submitted version.
