# Supplementary figures and images for: Multiparametric flow cytometry to characterize vaccine-induced polyfunctional T cell responses and T cell/NK cell exhaustion and memory phenotypes in mouse immuno-oncology models
Source: Front Immunol. 2023 Apr 6;14:1127896. doi: 10.3389/fimmu.2023.1127896 (PMC10115975; doi:10.3389/fimmu.2023.1127896)

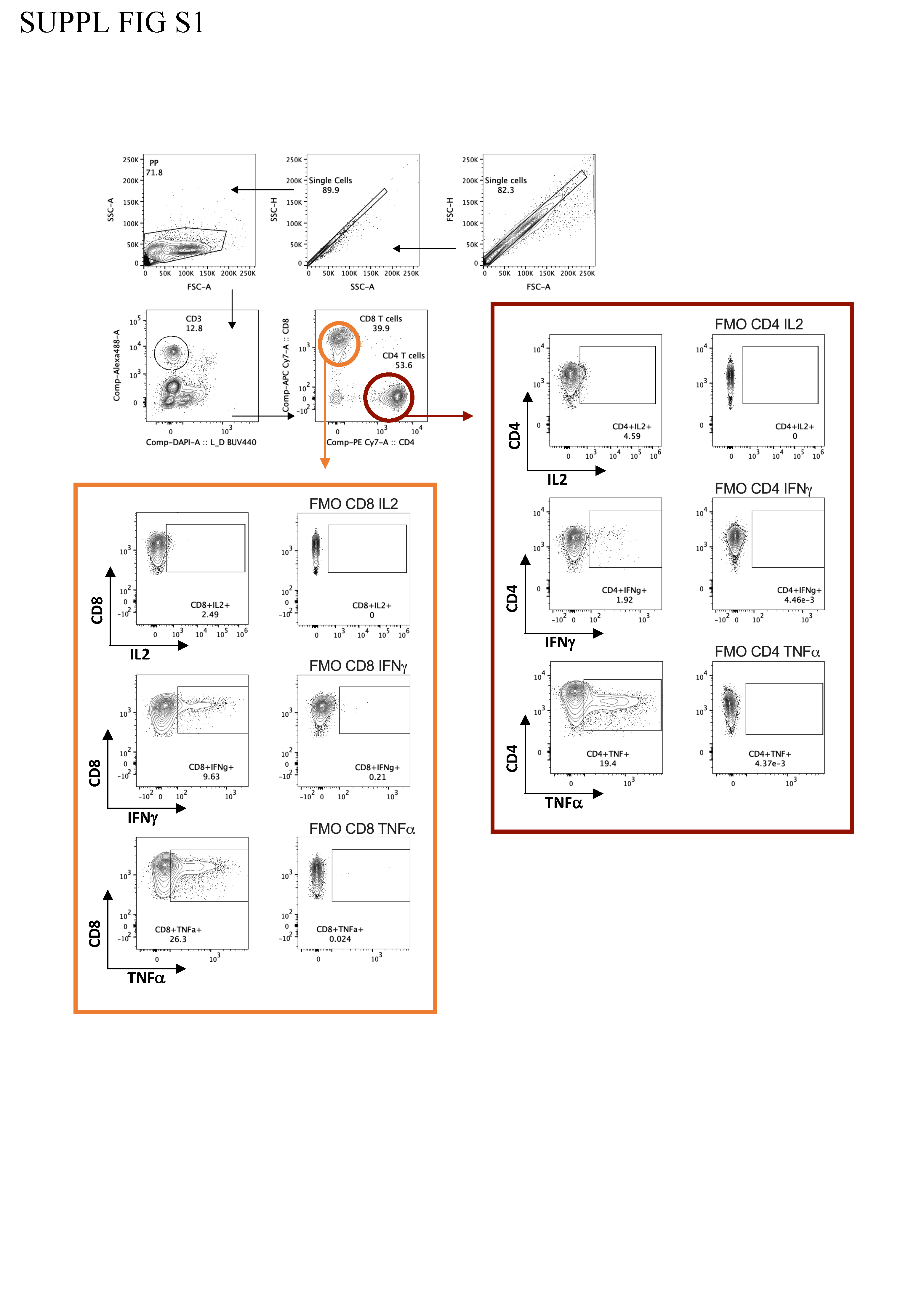

Supplement: Supplementary file 1 [file Image_1.tiff]

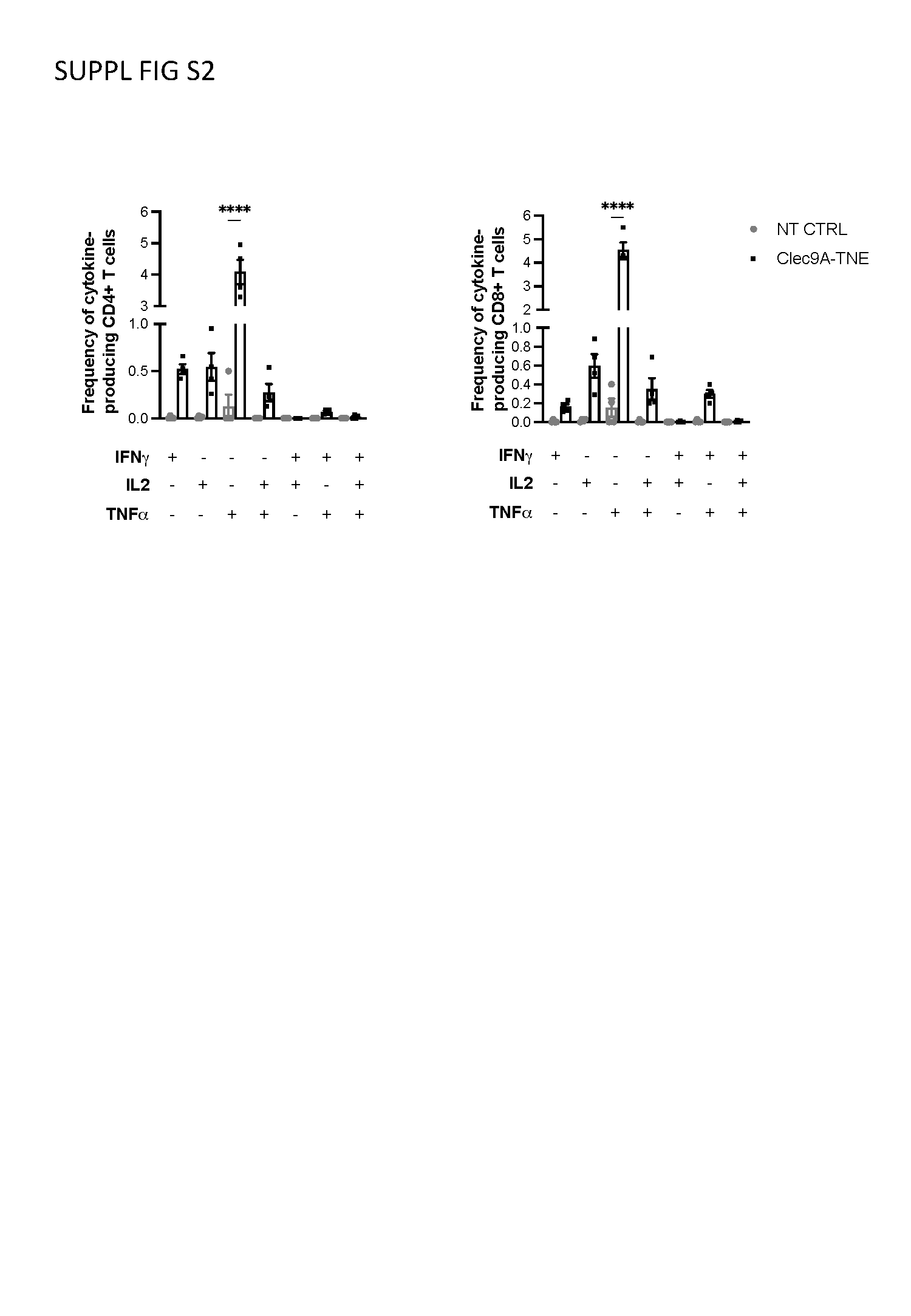

Supplement: Supplementary file 2 [file Image_2.tiff]

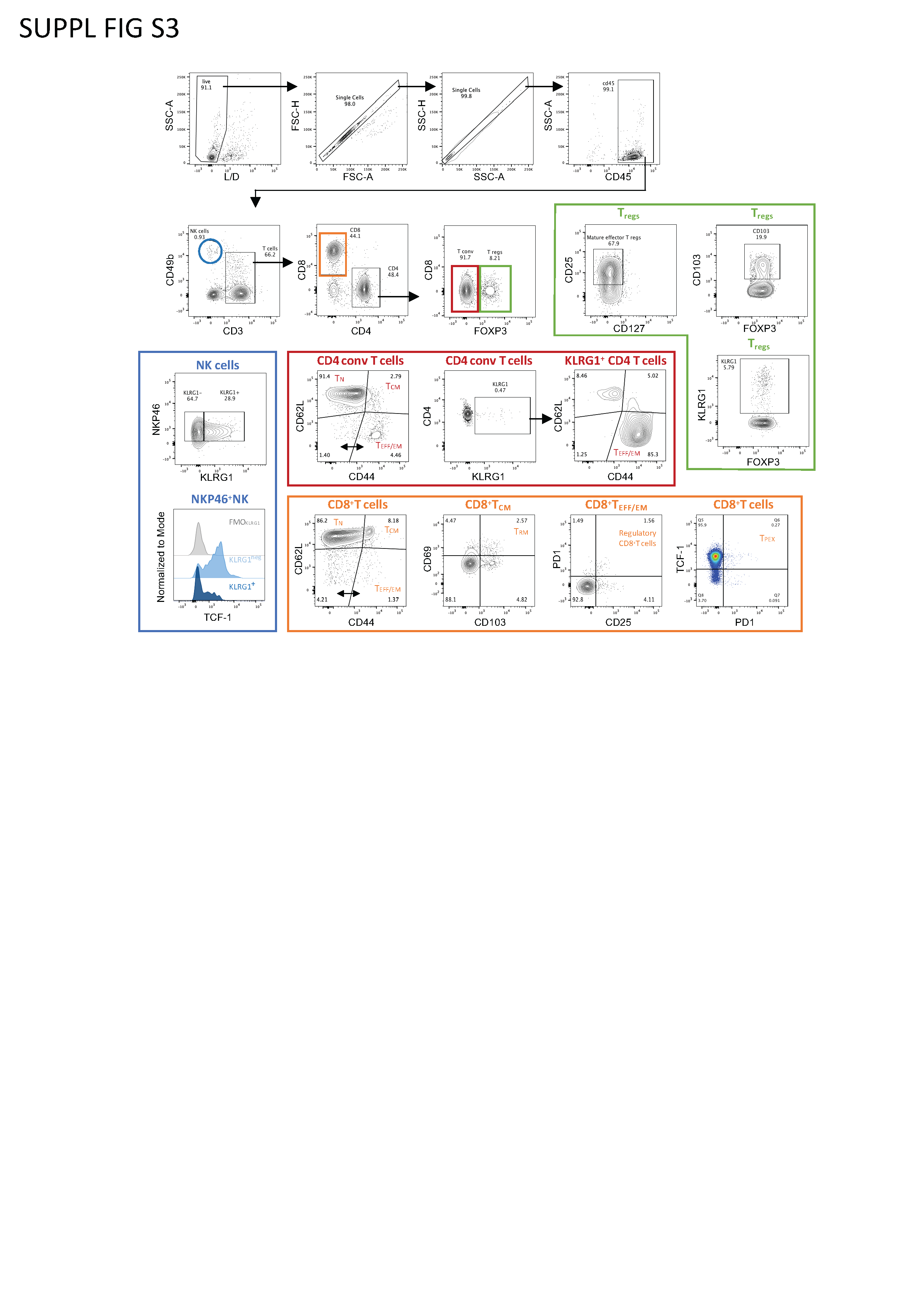

Supplement: Supplementary file 3 [file Image_3.tiff]

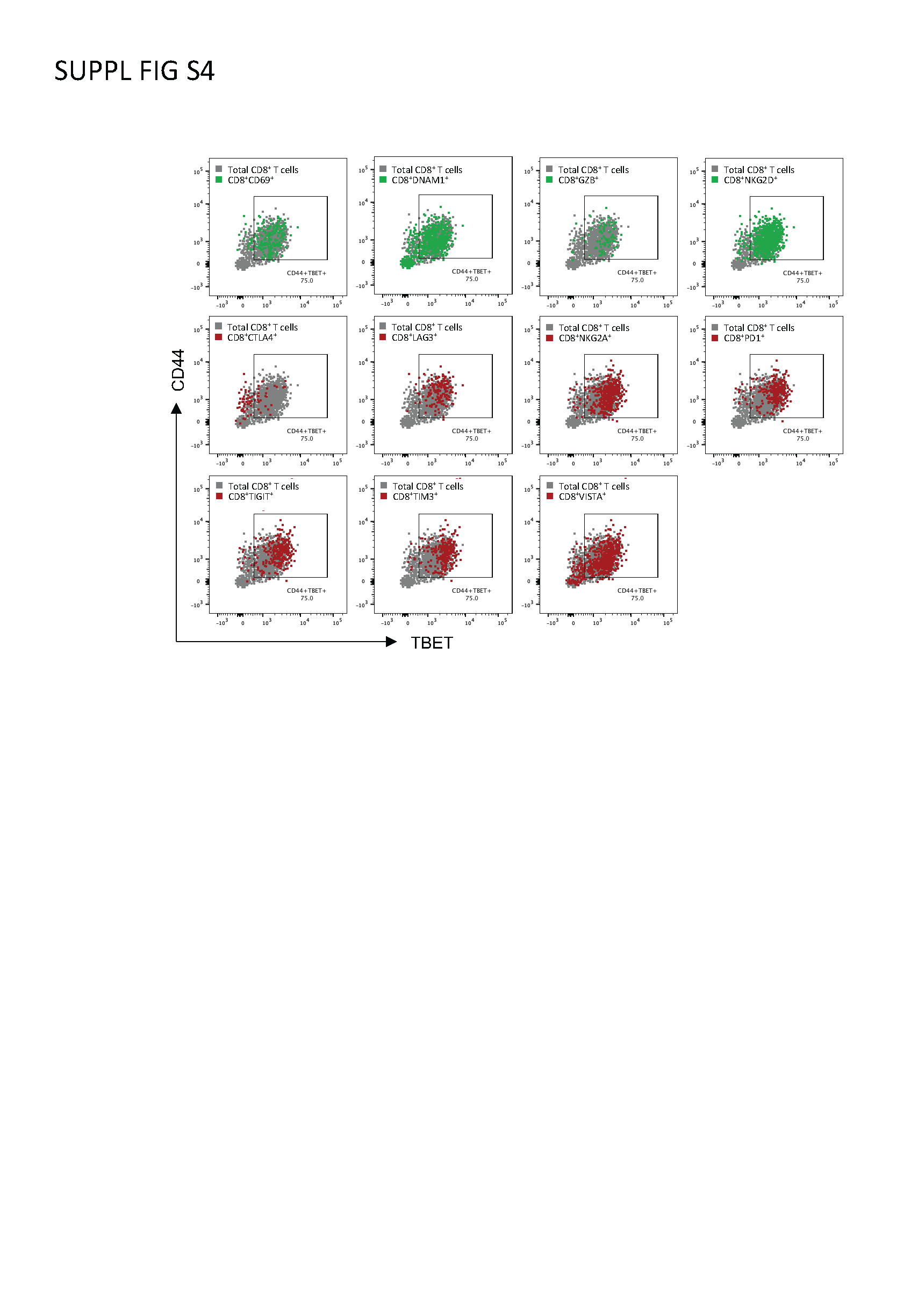

Supplement: Supplementary file 4 [file Image_4.tiff]

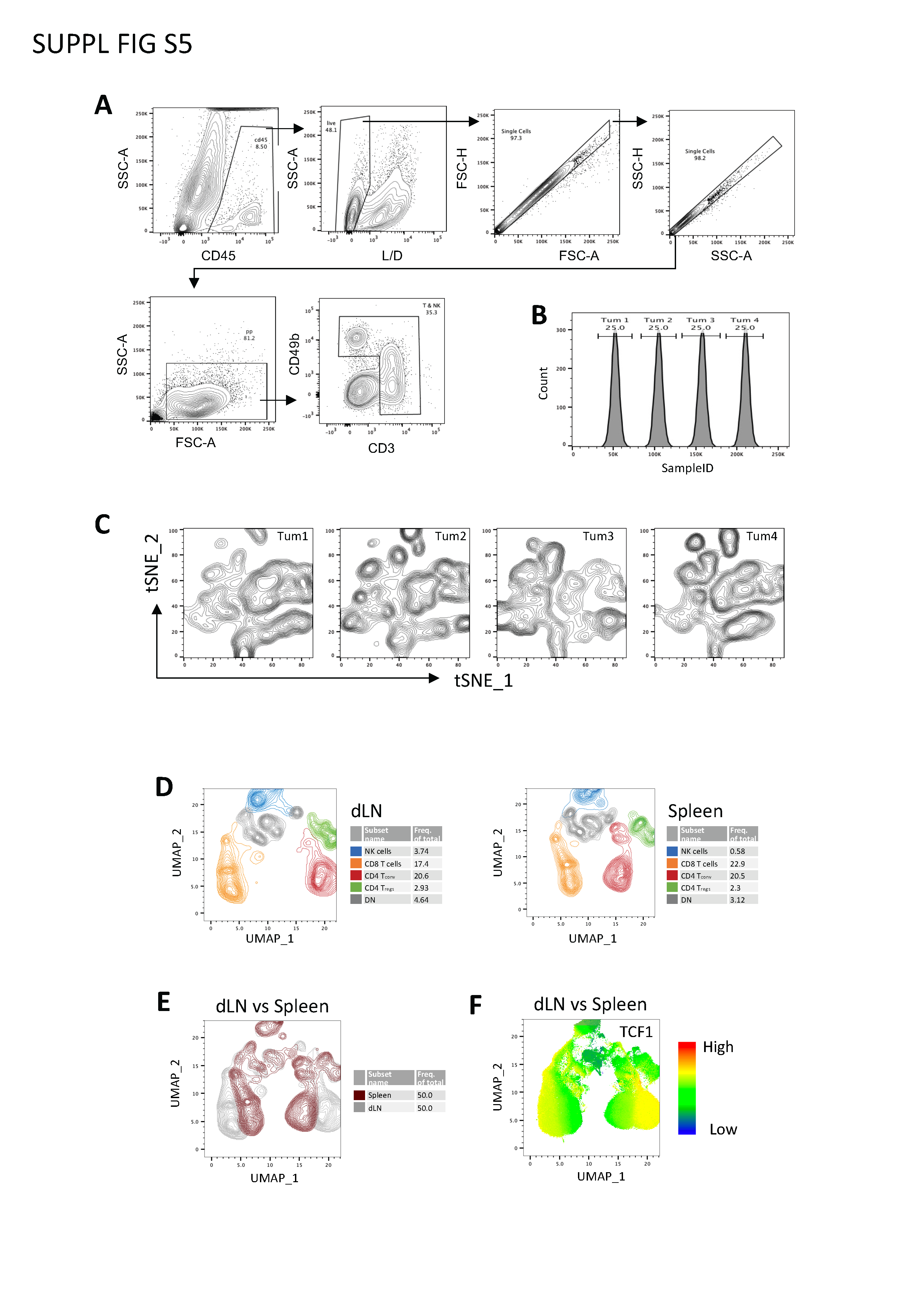

Supplement: Supplementary file 5 [file Image_5.tiff]

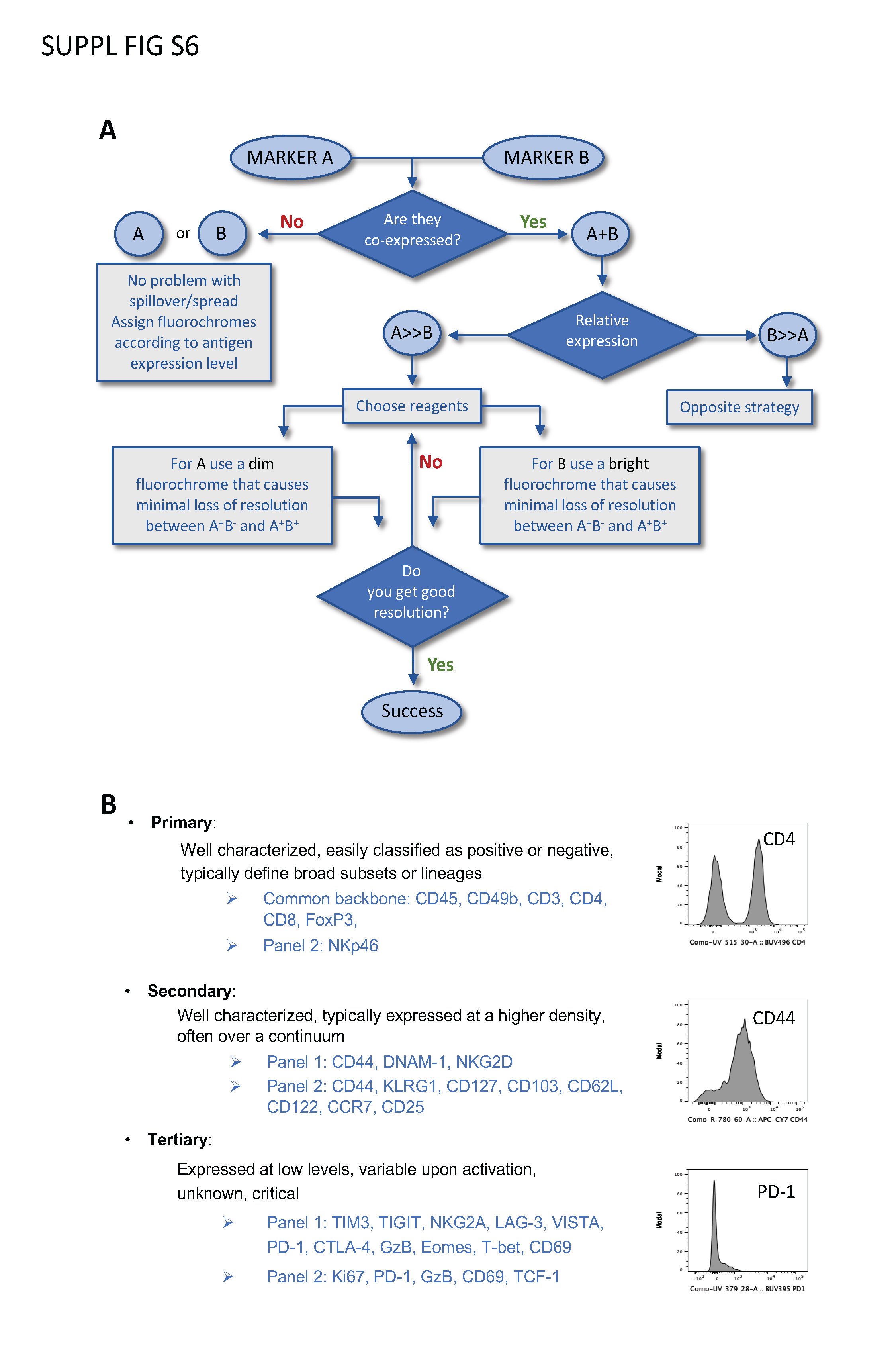

Supplement: Supplementary file 6 [file Image_6.tiff]

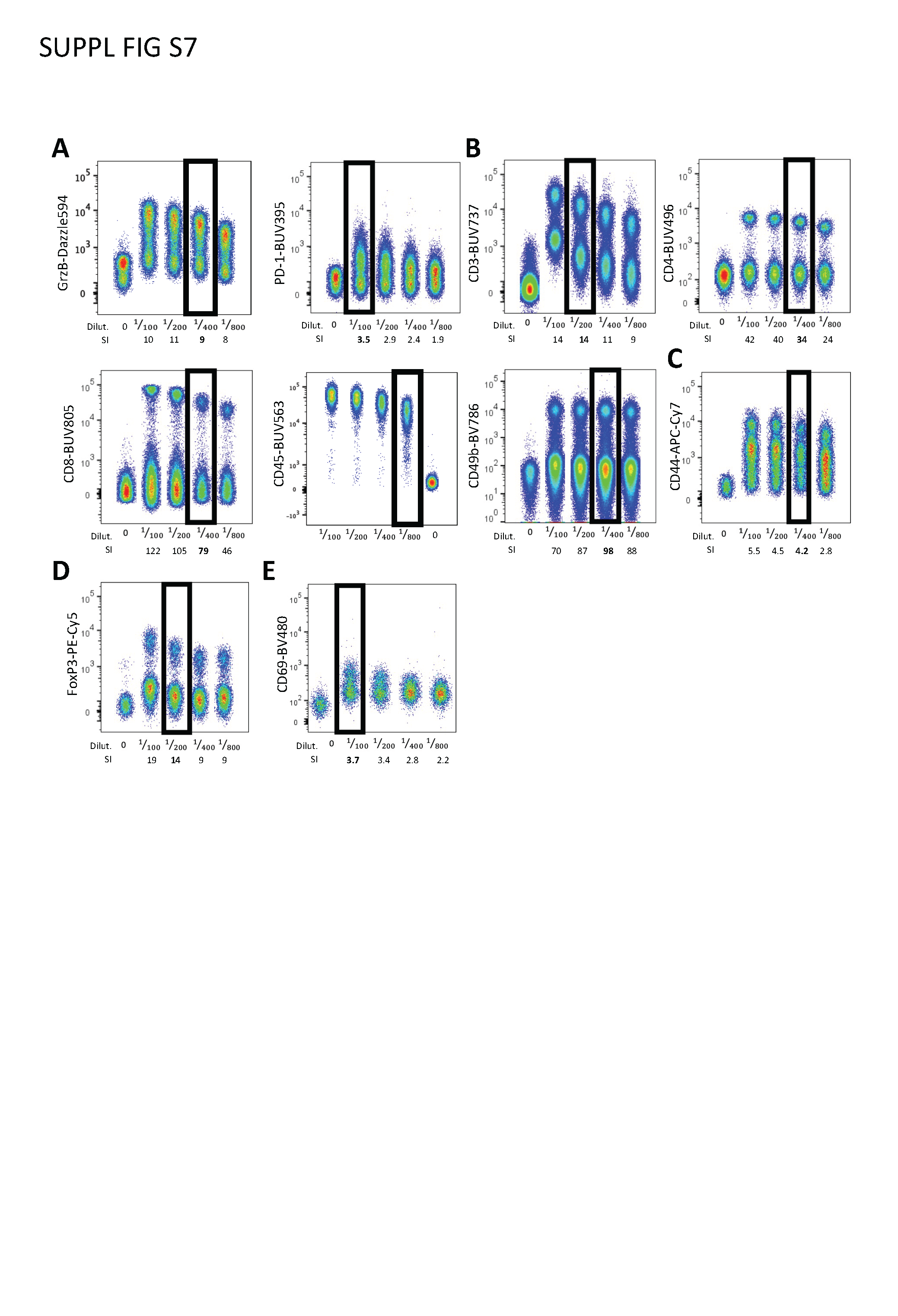

Supplement: Supplementary file 7 [file Image_7.tiff]

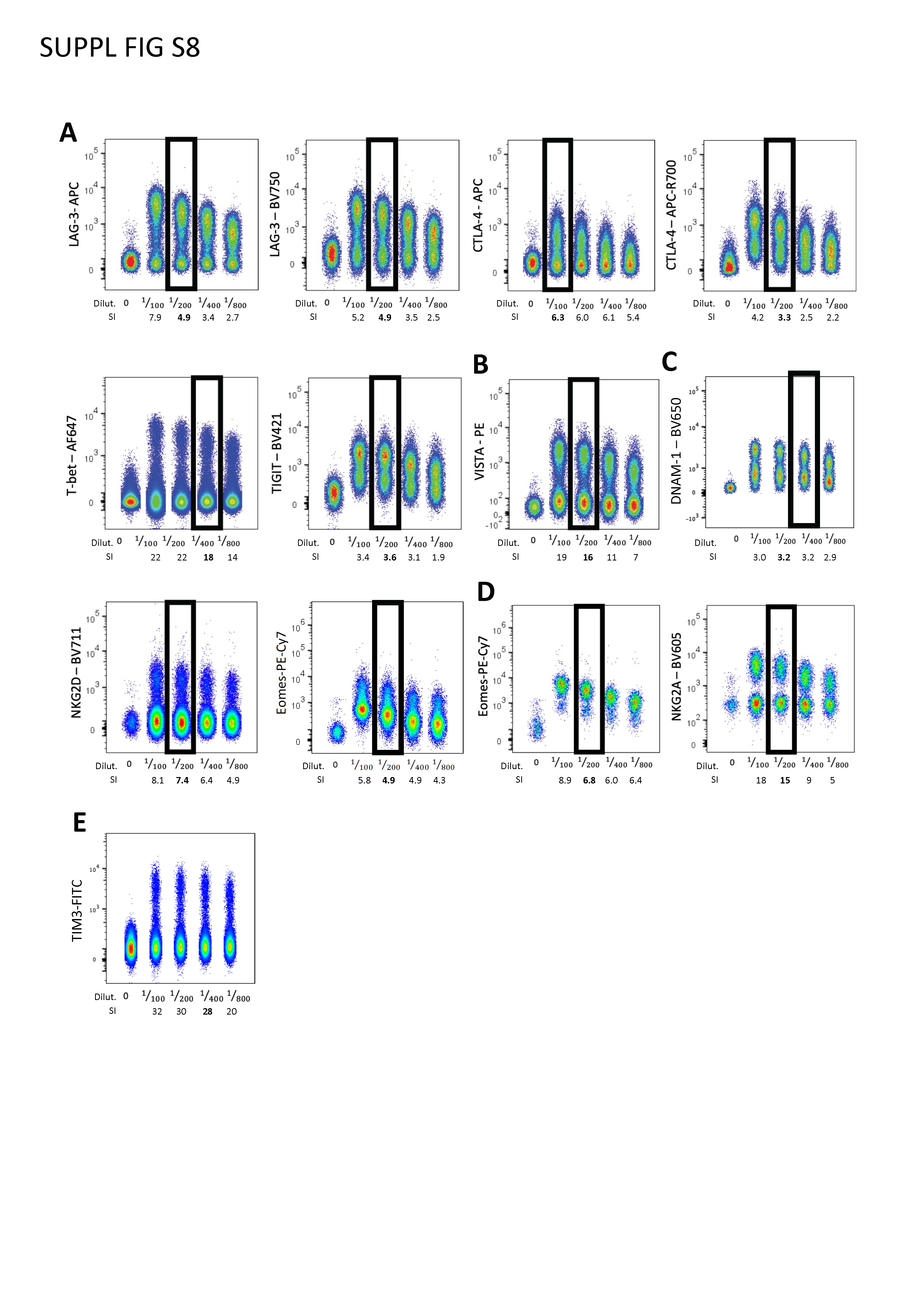

Supplement: Supplementary file 8 [file Image_8.tiff]

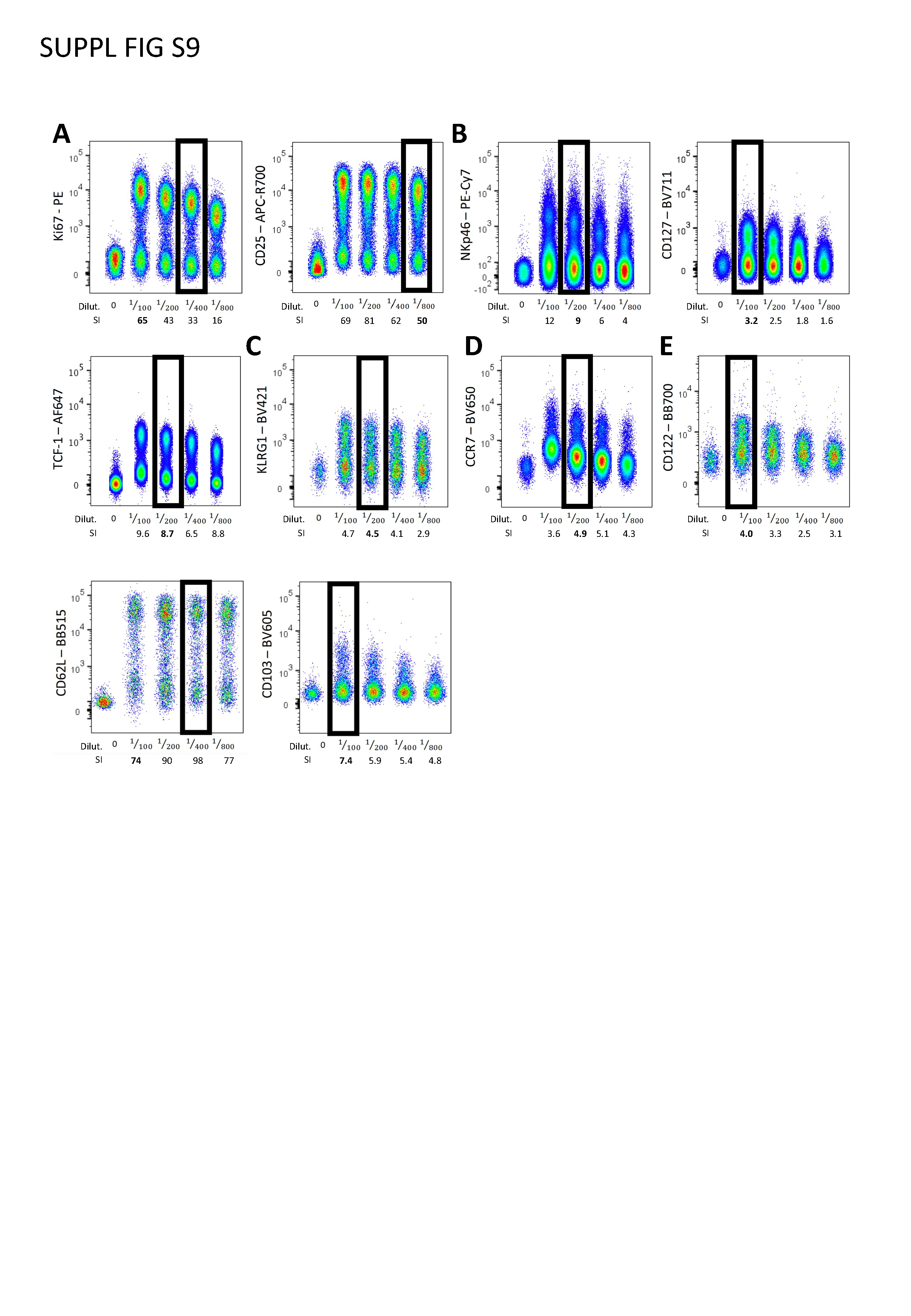

Supplement: Supplementary file 9 [file Image_9.tiff]

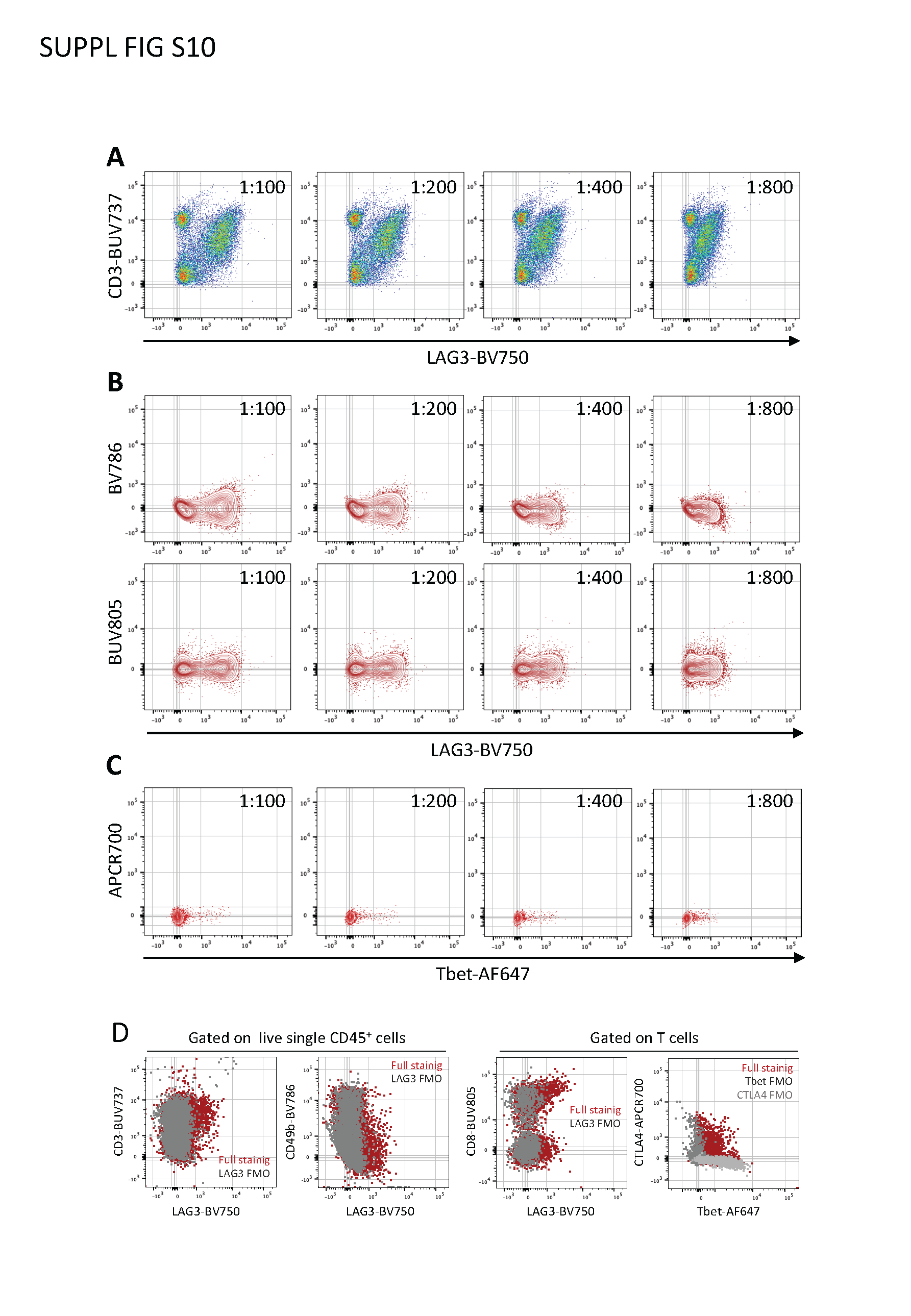

Supplement: Supplementary file 10 [file Image_10.tiff]

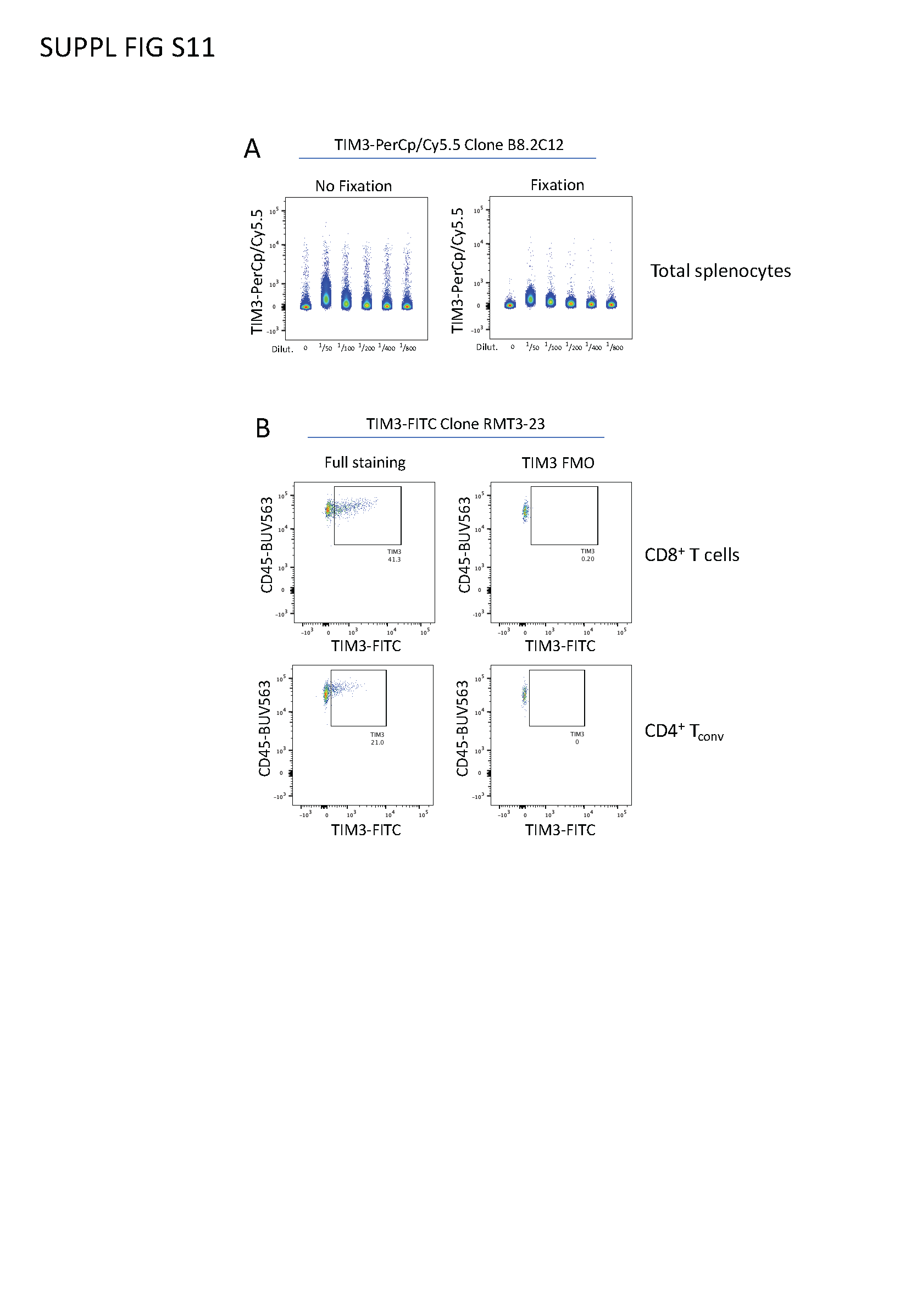

Supplement: Supplementary file 11 [file Image_11.tiff]

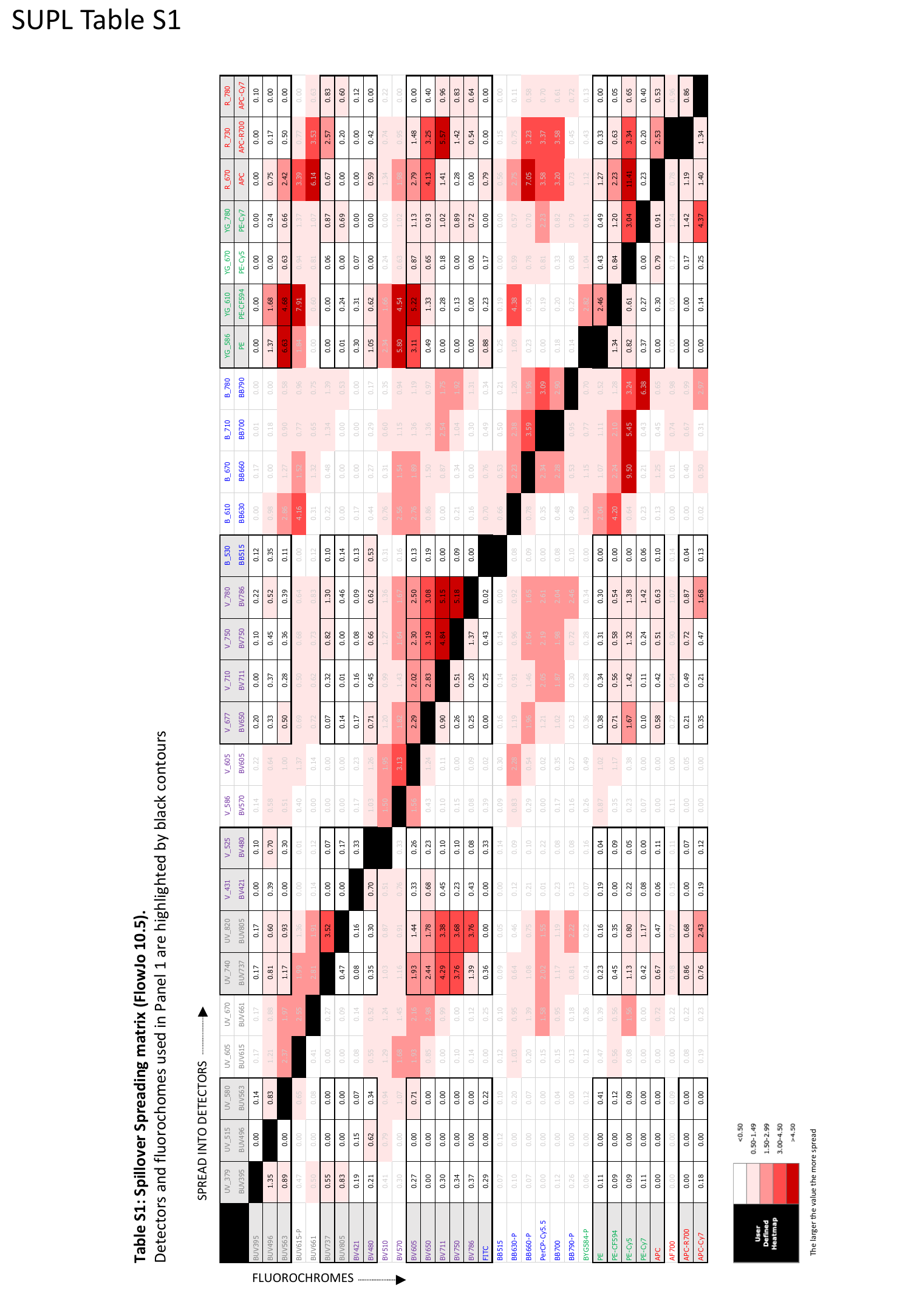

Supplement: Supplementary file 12 [file Image_12.tiff]

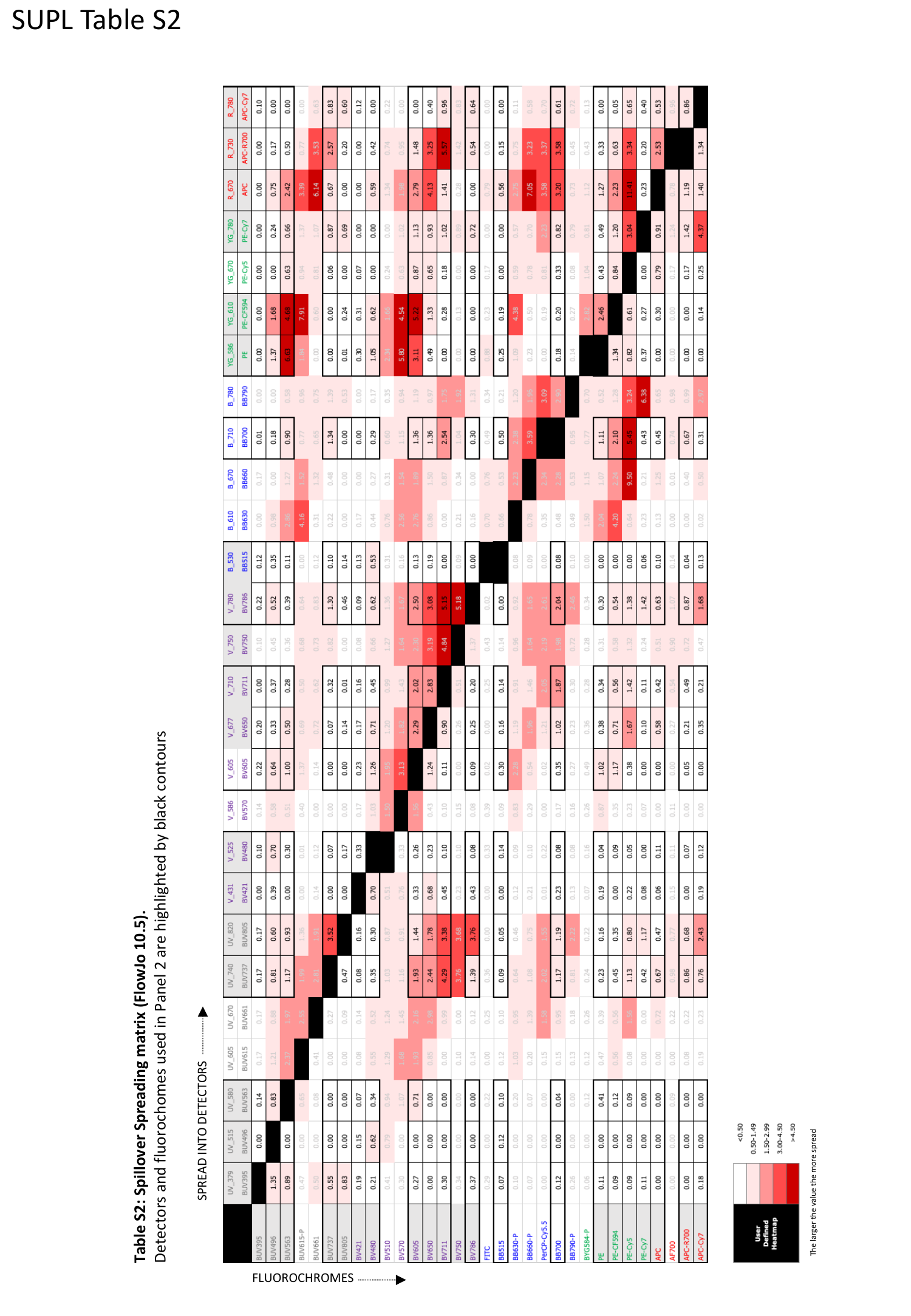

Supplement: Supplementary file 13 [file Image_13.tiff]
