# Supplementary material for: Multiparametric flow cytometry to characterize vaccine-induced polyfunctional T cell responses and T cell/NK cell exhaustion and memory phenotypes in mouse immuno-oncology models
Source: Front Immunol. 2023 Apr 6;14:1127896. doi: 10.3389/fimmu.2023.1127896 (PMC10115975; doi:10.3389/fimmu.2023.1127896)
Supplement: Supplementary file 14 [file DataSheet_1.docx]

**Multiparametric flow cytometry to characterize vaccine-induced polyfunctional T cell responses and T cell/NK cell exhaustion and memory phenotypes in mouse immuno-oncology models**

SUPPLEMENTARY INFORMATION

**21-COLOR PANEL DEVELOPMENT STRATEGY**

Panel 1 was conceived to extensively characterize the activation and exhaustion phenotypes of both T cells and NK cells primarily, but not exclusively, in the tumor microenvironment. Panel 2 was conceived to focus on maturation and memory/effector phenotypes of both T cells and NK cells in the periphery and in the tumor microenvironment. The two panels were designed with a common backbone of lineage and basic activation markers. This was done to allow versatility when addressing specific immunological questions relevant to immunotherapy in mouse cancer models. In addition, the use of shared markers beyond lineage across both panels allows for simultaneous assessment of activation, exhaustion and memory within defined cellular subsets (i.e. PD-1^+^ CD8^+^ T cells) (1-3).

The two panels were designed following the workflow described in Figure S6A. The following criteria were used to guide the panel design:

1. **Antigen classification**. Leucocyte antigens were categorized based upon their patterns of expression (Figure S6B). Primary markers were defined as those that are well characterized, expressed at high density, easily classified as positive or negative, and that typically define broad subsets or lineages. Secondary markers are also well characterized, typically expressed at a high density, and often with a continuum of expression. Tertiary markers are either expressed at lower levels, which may vary upon activation, or have still poorly defined expression levels (4).
2. **Fluorochrome availability and resolution ranking**. An instrument-specific fluorochrome brightness ranking was determined by BD staff as part of instrument characterization of the BD FACSymphony A5. Stain indices were calculated using LWB lymphocytes stained for human CD4 (clone SK3). Based on this ranking, bright dyes were preferentially assigned to dim antigens (low density, tertiary antigens).
3. **Spillover Spread matrix.** This matrix was generated for the BD FACSymphony A5 using freshly collected EDTA whole blood and anti-human CD4 on every fluorochrome. The spreading-error profile of each fluorochrome (Table S1 & S2), was accounted for in evaluating possible loss of resolution when co-expressed with other markers.

**Antibody titration and optimization**

After considering all the factors outlined above, the marker/fluorochrome associations described in Table 5 were chosen for Panel 1 & 2 respectively. Minimal optimization was required as described below. The highest concentration of antibody tested was a 1/100 dilution per sample and optimal antibody dilutions were chosen based on stain index calculations that also maintained the resolution of positive cells populations (Figures S7-S9). Experimental conditions were kept consistent during titration and final testing of full stained samples (i.e. in all titration tests, cells were fixed and permeabilised after surface staining).

**Staining Index (SI)** = MFI_Pos_ – MFI_neg_

2*SD_Neg_

**Panel optimisation**

In Panel 1, LAG-3, CTLA-4, T-bet and TIM-3 required additional optimization to achieve best antigen/fluorochrome association. Both LAG-3 and CTLA-4 were initially tested on APC as these markers are often not expressed at high levels. However, both markers were resolvable with alternative fluorochromes, BV750 and APC-R700, respectively (Figure S7A), and had acceptably low spread into related channels (Table S1). Importantly, the spread of LAG-3-BV750 into CD8-BUV805 did not compromise the resolution of CD8^+^Lag3^+^ double-positive T cells in tumor samples (Figure S10A, B & D). This allowed us to use APC for T-bet as this marker is often widely expressed and APC has a favorable spreading error profile (Table S1) allowing the identification of double-positive T-bet^+^CTLA-4^+^ T cells (Figure S10C & D). TIM-3 was initially tested using a PerCp/Cy5.5 conjugated antibody (clone B8.2C12, Biolegend); however, the signal did not survive fixation and due to the lack of relevant BB700-conjugated mAbs, this channel was left unused (Figure S11A). TIM-3 was instead moved to FITC (Figure S11B, clone RMT3-23, eBioscience), and although this fluorochrome is not particularly bright, distinct positive and negative populations were clearly resolvable in tumor samples. If the antibody becomes available, TIM-3 could be purchased on BB515 to provide greater separation, or a custom antibody could be acquired. No additional optimisation was required for Panel 2.

In our dissociated tumors, spleens and lymph nodes, there was acceptable spread amongst the different laser channel fluorochromes to allow resolution of all markers investigated by both panels. Nonetheless, with panels like these, where there is the potential for unpredictable co-expression of markers and changes in expression levels, care should be taken to titrate antibodies in each pre-clinical tumor model and include respective FMOs.

**References**

1. Wherry EJ, Kurachi M. Molecular and cellular insights into T cell exhaustion. Nat Rev Immunol (2015), 15(8):486-499.
2. Blank CU, Haining WN, Held W, Hogan PG, Kallies A, Lugli E, Lynn RC, Philip M, Rao A, Restifo NP, Schietinger A, Schumacher TN, Schwartzberg PL, Sharpe AH, Speiser DE, Wherry EJ, Youngblood BA, Zehn D. Defining 'T cell exhaustion'. Nat Rev Immunol (2019), 19(11):665-674.
3. Scott AC, Dündar F, Zumbo P, Chandran SS, Klebanoff CA, Shakiba M, Trivedi P, Menocal L, Appleby H, Camara S, Zamarin D, Walther T, Snyder A, Femia MR, Comen EA, Wen HY, Hellmann MD, Anandasabapathy N, Liu Y, Altorki NK, Lauer P, Levy O, Glickman MS, Kaye J, Betel D, Philip M, Schietinger A. TOX is a critical regulator of tumour-specific T cell differentiation. Nature (2019), 571(7764):270-274.
4. Mahnke YD, Roederer M. Optimizing a multicolor immunophenotyping assay. Clin Lab Med (2007), 27(3):469-85.

SUPPLEMENTARY FIGURE LEGENDS

**Figure S1. Manual gating strategy on blood cells (related to Figure 1).** Blood cells isolated from vaccinated and unvaccinated control mice were processed as described in methods for the polyfunctional assay. CD4^+^ T cells (*red*; CD3^+^CD4^+^) and CD8^+^ T cells (*orange*; CD3^+^CD8^+^) were analysed for the expression of IL2, IFNγ and TNFα according to the respective fluorescence minus one (FMO) control.

**Figure S2. Validation of the immunogenicity of the mSurvivin_53-67_ epitope in the Balb/c mouse background (related to Figure 1)**. By using the NetMHCpan - 4.1 prediction algorithm, we observed that the predicted binding affinity of the mSurvivin_53-67_ epitope to I-A^d^, I-E^d^ (Balb/c mice) is similar to that for I-A^b^ of C57BL/6 mice (not shown) for which the epitope has been so far used. To validate the immunogenicity of the mSurvivin_53-67_ epitope also in Balb/c cancer models, Balb/c mice (n=4) were injected i.v. with 100 μL Clec9a-TNE loaded with 25 μg of mSurvivin_53-67_ on day 0 and day 7. On day 14, spleens were collected and processed as described in Methods.  Cytokine expression in CD4^+^ and CD8^+^ T cells was assessed by flow cytometry and intracellular cytokine staining after *ex vivo* stimulation of splenocytes with the epitope. Significantly higher numbers of both CD8^+^ and CD4^+^ T cells expressing TNFα were observed in mice immunized with the epitope. A slight increase of borderline significance in CD8^+^ and CD4^+^ T cells expressing IFNγ, IL2 or cytokine combinations was also observed in immunized mice. Data are presented as mean values ±SEM.  (****) <0.0001, (Two-Way ANOVA).

**Figure S3. Manual gating on tumor draining lymph nodes stained with Panel 2 (related to Figure 2B).** Tumor draining lymph node isolated from mice bearing YUMM UV 1.7 melanomas were processed as described in methods. Immune cells were identified through exclusion of non-viable and CD45^−^ cells, followed by use of physical parameters to distinguish lymphocytes and exclude doublets. Main immune cell subsets were defined as follows: natural killer cells (*blue*; NK; CD3^−^CD49b^+^); conventional CD4^+^ T cells (*red*; CD4^+^ T_conv_; CD3^+^CD4^+^FoxP3^−^); regulatory CD4^+^ T cells (*green*; T_reg_; CD3^+^CD4^+^FoxP3^+^) and CD8^+^ T cells (*orange*, CD3^+^CD8^+^). Examples of additional subpopulations are also indicated as follow: mature and differentiated NK cells (NKp46^+^KLRG1^+^); Naïve (T_N_: CD62L^+^CD44^-^), central memory (T_CM_: CD62L^+^CD44^+^) or effector/effector memory (T_EFF/EM_: CD62L^-^CD44^+^) CD4^+^ and CD8^+^ T cells; Cytotoxic CD4^+^ T cells (CD4^+^FOXP3^-^KLRG1^+^); Tissue resident memory CD8^+^ T cells (T_RM:_ CD69^+^CD103^+^CD8^+^ T_CM_); regulatory CD8^+^ T cells (PD-1^+^CD25^+^CD8^+^ T_EEF/EM_); precursors of exhausted T cells (PD-1^+^TCF1^+^CD8^+^ T_PEX_).

**Figure S4. Activation versus exhaustion markers (related to Figure 3).** YUMM1.7 UV tumors were manually dissociated and stained with Panel 1 as described in Methods. CD8^+^ T cells were gated as described in Figure 2A. Representative dot plots from YUMM1.7 UV tumors are shown. *Gray*: total CD8^+^ T cells with a gate on CD44^+^Tbet^+^ effector cells; *green*: overlay of CD8^+^ T cells positive for the indicated activation marker; *red*: overlay of CD8^+^ T cells positive for the indicated immune checkpoint inhibitors.

**Figure S5. High-dimensional data analysis with Panel 1 & 2 (related to Figures 5 & 6). (A)** Gating strategy to define a single gate including T and NK cells. Tumor infiltrating immune cells from YUMM UV 1.7 tumors were identified through exclusion of non-viable and CD45^−^ cells; followed by use of physical parameters to distinguish lymphocytes and exclude doublets. Last, a single gate was designed to exclude double negative CD3^-^CD49b^-^ cells. The same gating strategy was used to obtain a T/NK single gate with spleens and draining lymph nodes **(B)** Histograms on concatenated T and NK cells from 4 independent YUMM UV 1.7 tumors used to generate the tSNE in Figure 2. Same approach was used for spleen and draining lymph node concatenates **(C)** Individual tSNE plots on T and NK cells from 4 independent YUMM UV 1.7 tumors. **(D-F)** Spleen samples as in Figure 2B**.** Draining lymph nodes were isolated from the same mice and processed as described in methods**.**  **(D)** UMAP plot on concatenated T and NK cells from 3 lymph nodes (left) and 3 spleens (right) isolated from mice bearing YUMM UV 1.7 tumours. The plot shows overlayed coloured manual gates for CD8^+^ T cells (*orange*), CD4^+^ T_conv_ cells (*red*), CD4^+^ T_reg_ cells (*green*), NK cells (*blue*), and double negative (CD4^-^CD8^-^) T cells (*grey*). Contour plots reveal cell density and main subpopulations. (**E**) Overlay of the same UMAPs as in the top plots. *Grey*: draining lymph nodes; *Brown*: splenocytes. **(F)** Visualization of the expression phenotype for TCF1 across all T and NK cell clusters in UMAP. Marker expression intensity is indicated by the scale bar at the bottom of the plots where blue is low and red is high.

**Figure S6. Multiparametric Panel Design. (A)** Panel Design Workflow for Optimal Fluorochrome-Antigen Matching. **(B)** Antigen classification based on expression levels and expression profile. <https://www.bdbiosciences.com/en-ca/resources/panel-design#Videos>.

**Figure S7. Titration of the common backbone antibodies. (A)** Whole splenocytes from a naïve C57Bl/6 mouse were stimulated for 72 hours with plate bound anti-CD3 antibody (1μg/ml) and soluble anti-CD28 (1μg/ml). Unstimulated splenocytes were plated at the same time and a 1:2.5 ratio of unstimulated to stimulated cells was mixed and stained with FVSUV440 live/dead for antibody titration. **(B)** Splenocytes from a naïve C57Bl/6 were stained with FVSUV440 live/dead and antibody titration was performed on total lymphocytes (identified using physical parameters). **(C,D)** Splenocytes from a naïve C57Bl/6 mouse were stained with FVSUV440 live/dead, anti-CD3, anti-CD4. Antibody titration was performed on **(C)** T cells (CD3^+^) and **(D)** CD4^+^ T cells. **(E)** Bone marrow was harvested from a naïve C57Bl/6 mouse and antibody titration was performed on T cells. All samples were fixed with the eBioscience FoxP3 kit prior to acquisition to replicate experimental conditions. Concatenated flow cytometry plots show antibody titration dilutions (Dilut.) ranging from 1/100 to 1/800, with an unstained control (0), for fluorochrome conjugated antibodies. SI = staining index. Black bars indicate optimal concentration.

**Figure S8. Titration of antibodies specific for Panel 1. (A)** Whole splenocytes from a naïve C57Bl/6 mouse were stimulated for 72 hours with plate bound anti-CD3 antibody (1 μg/ml) and soluble anti-CD28 (1 μg/ml). Unstimulated splenocytes were plated at the same time and a 1:2.5 ratio of unstimulated to stimulated cells was mixed and stained with FVSUV440 live/dead for antibody titration. **(B)** Splenocytes from a naïve C57Bl/6 were stained with FVSUV440 live/dead and antibody titration was performed on total lymphocytes (identified using physical parameters). **(C,D)** Splenocytes from a naïve C57Bl/6 were stained with FVSUV440 live/dead, anti-CD3, anti-CD49b, and anti-CD4. Antibody titration was performed on **(C)** T cells (CD3^+^), **(D)** NK cells (CD49b^+^). (**E**) A lymph node from a 4T1.2 tumour bearing mouse was manually dissociated for titration of TIM-3–FITC on CD45^+^CD11B^-^ cells. Concatenated flow cytometry plots show antibody titration dilutions (Dilut.) ranging from 1/100 to 1/800, with an unstained control (0), for fluorochrome conjugated antibodies. All samples were fixed with the eBioscience FoxP3 kit prior to acquisition to replicate experimental conditions. SI = staining index. Black bars indicate optimal concentration.

**Figure S9. Titration of antibodies specific for Panel 2. (A)** Whole splenocytes from a naïve C57Bl/6 mouse were stimulated for 72 hours with plate bound anti-CD3 antibody (1 μg/ml) and soluble anti-CD28 (1 μg/ml). Unstimulated splenocytes were plated at the same time and a 1:2.5 ratio of unstimulated to stimulated cells was mixed and stained with FVSUV440 live/dead for antibody titration. **(B)** Splenocytes from a naïve C57Bl/6 were stained with FVSUV440 live/dead and antibody titration was performed on total lymphocytes (identified using physical parameters). **(C,D)** Splenocytes from a naïve C57Bl/6 were stained with FVSUV440 live/dead, anti-CD3, anti-CD49b, and anti-CD4. Antibody titration was performed on **(C)** T cells (CD3^+^), **(D)** NK cells (CD49b^+^). (**E**) Bone marrow was harvested from a naïve C57Bl/6 mouse and antibody titration was performed on T cells. Concatenated flow cytometry plots show antibody titration dilutions (Dilut.) ranging from 1/100 to 1/800, with an unstained control (0), for fluorochrome conjugated antibodies. All samples were fixed with the eBioscience FoxP3 kit prior to acquisition to replicate experimental conditions. SI = staining index. Black bars indicate optimal concentration.

**Figure S10. LAG-3-BV750 and Tbet-AF647 spread.** Splenocytes from a naïve C57Bl/6 mouse were stimulated as in Figure S6A. Unstimulated splenocytes were plated at the same time and a 1:2.5 ratio of unstimulated to stimulated cells was mixed and stained with FVSUV440 live/dead (1:1000), CD3-BUV737 (1:200) and either LAG-3-BV750, or Tbet-AF647 (1:100, 1:200, 1:400, and 1:800). Based on the Spillover Spreading matrix in the online Table 2, spreading of LAG-3-BV750 into BUV737 **(A)** or BV786 and BUV805 **(B)**; and of Tbet-AF647 into APC-R700 **(C)** were verified for each dilution of the LAG-3 and T-bet antibodies. **(D)** YUMM1.7UV tumors were enzymatically digested and stained with Panel 1. Representative dot plots for the indicated markers with fully stained samples (*red*) and relative FMO controls (*dark and light grey*) overlays. All samples were fixed with the eBioscience FoxP3 kit prior to acquisition.

**Figure S11: Anti-TIM-3 titration on exhausted splenocytes. (A)** Exhausted splenocytes were obtained from C57Bl/6 mice that had been injected with the chronic clone-13 strain of lymphocytic choriomeningitis virus (LCMV) post-CD4 T cells depletion using the GK1.5 antibody. Exhausted splenocytes were stained with FVSUV440 live/dead for TIM-3-Per-Cp-Cy5.5 titration (clone B8.2C12). Half samples were fixed with the eBioscience FoxP3 kit prior to acquisition to replicate experimental conditions and half were left unfixed. Concatenated flow cytometry plots show antibody titration dilutions (Dilut.) ranging from 1/100 to 1/800, with an unstained control (0), for fluorochrome conjugated antibodies. **(B)** Full staining with Panel 1 on YUMM1.7UV tumours using TIM-3-FITC (clone RMT3-23). Gating of TIM3^+^ cells on tumour infiltrating (YUMM1.7UV tumours) CD8^+^ T cells (top) and T_conv_ cells (bottom) based on corresponding FMO controls.
